# Tissue‐selective regulation of protein homeostasis and unfolded protein response signalling in sporadic ALS

**DOI:** 10.1111/jcmm.15170

**Published:** 2020-04-23

**Authors:** Luigi Montibeller, Li Yi Tan, Joo Kyung Kim, Praveen Paul, Jacqueline de Belleroche

**Affiliations:** ^1^ Neurogenetics Group Department of Brain Sciences Faculty of Medicine Imperial College London London UK

**Keywords:** amyotrophic lateral sclerosis (ALS), ERAD, frontotemporal lobar dementia (FTLD), heat shock response (HSR), PDI, unfolded protein response (UPR)

## Abstract

Amyotrophic lateral sclerosis (ALS) is a disorder that affects motor neurons in motor cortex and spinal cord, and the degeneration of both neuronal populations is a critical feature of the disease. Abnormalities in protein homeostasis (proteostasis) are well established in ALS. However, they have been investigated mostly in spinal cord but less so in motor cortex. Herein, we monitored the unfolded protein (UPR) and heat shock response (HSR), two major proteostasis regulatory pathways, in human post‐mortem tissue derived from the motor cortex of sporadic ALS (SALS) and compared them to those occurring in spinal cord. Although the UPR was activated in both tissues, specific expression of select UPR target genes, such as PDIs, was observed in motor cortex of SALS cases strongly correlating with oligodendrocyte markers. Moreover, we found that endoplasmic reticulum‐associated degradation (ERAD) and HSR genes, which were activated predominately in spinal cord, correlated with the expression of neuronal markers. Our results indicate that proteostasis is strongly and selectively activated in SALS motor cortex and spinal cord where subsets of these genes are associated with specific cell type. This study expands our understanding of convergent molecular mechanisms occurring in motor cortex and spinal cord and highlights cell type–specific contributions.

## 1 INTRODUCTION

Amyotrophic lateral sclerosis (ALS) is the most common adult‐onset neuromuscular disorder, which selectively targets motor neuron (MN) populations in the spinal cord, brain stem and motor cortex and leads to death due to respiratory failure, typically within 2‐5 years of symptom onset.^1^ Degeneration of spinal cord MNs causes atrophy and muscle wasting, while the spasticity reflects the sclerosis of the lateral and ventral corticospinal tracts arising from the motor cortex^1^, ^2^ However, although MN degeneration in both spinal cord and motor cortex is an essential component of the disease and necessary to confirm diagnosis of ALS^1^, ^3^ the nature of the interactions between these two systems in initiating and promoting disease progression has not been resolved. Neuropathologically, both cortical tissue and spinal tissue are characterized by the presence of TDP‐43^+ve^ inclusions that are found in 97% of ALS cases. These inclusions have been found also in cases of frontotemporal degeneration (FTLD), which share clinical, genetic and histopathological features with ALS^1^ For these reasons, FTLD and ALS have been considered as disease manifestations of the same clinicopathological spectrum. In particular, related pathological features are evident in advanced disease stages, when TDP‐43 pathology and cortical tissue changes of FTLD and ALS can be similarly abundant due to more profound degenerative changes.^4^, ^5^ The accumulation of TDP‐43^+ve^ inclusions in these disorders is suggestive of defects in protein homeostasis (proteostasis). To cope with this, the unfolded protein response (UPR) and heat shock response (HSR) are two of the major signalling pathways aiming at restoring proteostasis.^6^, ^7^

The UPR emanates from the endoplasmic reticulum (ER) and is transduced by three ER‐resident transmembrane proteins, namely IRE1α, ATF6α and PERK, whose signalling aims at restoring ER protein homeostasis (proteostasis) through transcriptional and post‐transcriptional mechanisms.^7^ In ALS patients’ tissues, the UPR is activated predominately in the spinal cord,^8^ where we have observed a profound activation of IRE1α‐XBP1s and ATF6 signalling arms and their target genes mainly involved in protein degradation and protein folding processes.^9^, ^10^, ^11^

The HSR is orchestrated by the transcription factor called heat shock factor 1 (HSF1), which is activated during acute proteostatic stress and is capable of activating the transcription of genes coding for chaperones, known as heat shock proteins (HSPs), to protect the cell from the accumulation of aberrantly folded proteins.^12^ HSF1 and its target genes, such as *HSPB1, HSPB8* or DNAJB1, are up‐regulated in SOD1 mice where they enhance MN survival.^13^ Although HSF1 has been detected in spinal cord MN,^14^, ^15^ its activation and cellular localization in the motor cortex of ALS patients have not been characterized yet. Although the contribution of the motor cortex and spinal cord degeneration represents a key aspect in the disease pathogenesis, a clear understanding of the molecular and cellular mechanisms occurring concurrently in these two critical CNS regions is still missing.

Here, we compared the activation of UPR and HSR in the spinal cord, with those occurring in the motor cortex of ALS cases. We found that the UPR was activated in both spinal cord and motor cortex, which was characterized by a specific up‐regulation of PDI‐encoding genes, while the HSR was predominately dysregulated in the spinal cord. We also identified a strong correlation between UPR activation and oligodendrocyte markers in the motor cortex and with neuronal markers in the spinal cord. This study expands our understanding of convergent and divergent molecular mechanisms occurring in these two brain regions and highlights the regional and cellular proteostasis alteration in ALS.

## 2 MATERIALS AND METHODS

### 2.1 Patients and tissue sample preparation

Motor cortex samples were available for 23 post‐mortem necropsies and comprised 10 SALS cases with a median age at death of 66.5 years (range 48‐82 years) and median post‐mortem delay of 14 hours (range 7.5‐60 hours) and 13 control cases with a median age at death of 67 years (range 20‐94 years) and median post‐mortem delay of 10.75 hours (range 3‐35 hours). All the selected cases were characterized by upper and lower motor neuron degeneration. Frozen dorsolateral prefrontal cortex and temporal cortex tissues were obtained from 40 patients: 20 were healthy controls with only ageing‐related changes and 20 were from frontotemporal lobar degeneration (FTLD) cases. All samples were neuropathologically characterized by the presence of TDP‐43^+ve^ inclusions and lacked hexanucleotide repeat expansions in *C9ORF72*. Characteristics and clinical information of the spinal cord, frontal and temporal cortex samples including gender, age at death and post‐mortem delay (PMD) was reported previously.^9^, ^16^ This study was approved by the Riverside Research Ethics Committee and was carried out according to their guidelines. All clinical diagnoses were confirmed neuropathologically at post‐mortem. More details can be found in SI Materials and Methods.

### 2.2 Selection of cell type markers

Three main markers were considered for each cell type: GFAP for astrocyte,^17^, ^18^ MOG for oligodendrocytes^19^, ^20^ and ENO2 for neuronal population.^21^, ^22^, ^23^ The specificity of these markers was also confirmed by single‐cell studies.^23^, ^24^, ^25^ More details can be found in SI Materials and Methods.

### 2.3 mRNA extraction, RNA quality assessment and quantitative PCR (qPCR)

mRNA was extracted, and RNA quality was assessed as previously described.^9^ Briefly, RNA was extracted from motor cortex, spinal cord, frontal and temporal cortex samples following Direct‐zol RNA mini prep (Zymo Research) protocol. RNA purity and integrity for all samples was assessed by using multiple well‐established methods***.*** Quantitative PCR was performed using the Power Up™ SYBr™ Green Master Mix (Thermo Fisher Scientific). Primer sequences and temperatures utilized for real‐time PCR analysis are previously reported^9^ or listed in the Table S2. More details can be found in SI Materials and Methods.

### 2.4 Western blot analyses

Sections from frozen tissue blocks were prepared as previously described.^9^, ^16^ Blotting was carried out using conditions specified for the antibodies following the procedure reported in.^9^ The list of antibodies used, and more details can be found in SI Materials and Methods.

### 2.5 Immunohistochemical analyses

Immunohistochemistry was carried on paraffin sections from the same cases used for mRNA analysis in frozen samples. These cases consisted of 10 controls (7 male and 3 female), mean age of 73.6 ± 4.05 years with a post‐mortem delay of 36.9 ± 4.88 (Mean ± SEM) hours and 10 FTLD cases (positive for TDP‐43 inclusions but lacking C9ORF72 hexanucleotide expansions) (6 males and 4 females) mean age of 80.9 ± 3.21 (Mean ± SEM) years with a post‐mortem delay of 29.85 ± 4.01 hours. The list of antibodies used, and more details can be found in SI Materials and Methods.

### 2.6 Statistical analyses

Statistical analyses were performed using GraphPad Prism 7 software (GraphPad). Results were expressed as mean ± SEM unless otherwise indicated. All statistical analyses were performed using two‐tailed Student's *t* test after checking for normality of the data. For the correlation analyses, Pearson's correlation was used when data were normally distributed, and Spearman's correlation was used when data were not sampled from a Gaussian distribution. Benjamini‐Hochberg FDR test was used to adjust *P* values to correct for type I errors. A *P* value of <.05 was considered significant. More details can be found in SI Materials and Methods.

## 3 RESULTS

### 3.1 Common features between the spinal cord and motor cortex derived from SALS cases in the expression of UPR genes at the mRNA and protein level

**Figure 1:**
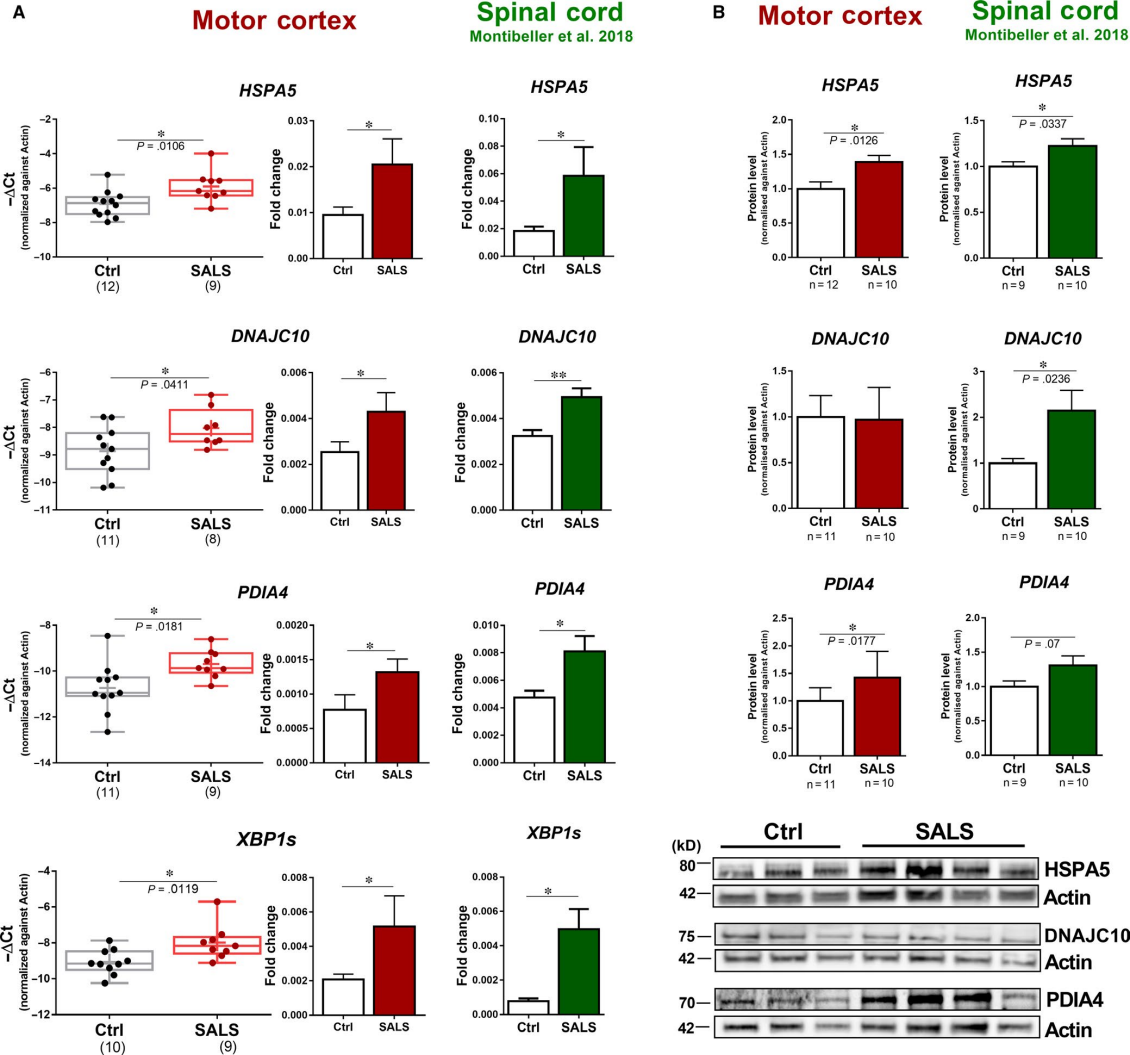
Common gene and protein expression in the motor cortex and spinal cord in SALS cases. A, mRNA expression analysis of XBP1s, HSPA5, PDIA4 and DNAJC10 in the motor cortex of healthy individuals (Ctrl, black) and sporadic cases of amyotrophic lateral sclerosis (SALS, red). Box plots and bar plots are representations for the same samples. Median, maximum and minimum values were used to represent the data in box and whiskers plots; mean was shown as ‘+’inside the box. Means and SEMs were used to represent the data in the bar plot. The dots represent individual samples. B, Western blot analyses are shown for HSPA5, DNAJC10 and PDIA4 detected in the motor cortex of healthy individuals (Ctrl, white) and SALS cases (SALS, red). Quantitative analyses of each protein were performed by densitometry of the relative bands in control and SALS cases. Values were obtained using beta‐actin as a reference gene. Means and SEMs were used to represent the data. C, Representative Western blots are shown for HSPA5, DNAJC10 and PDIA4 in the motor cortex. mRNA and protein expression in spinal cord samples (SALS, green) were obtained from Montibeller and de Belleroche.^8^ The numbers under the graphs represent the number of samples analysed. SALS, sporadic amyotrophic lateral sclerosis; Ctrl, control. According to D’Agostino and Pearson normality test, all data are normally distributed. Unpaired *t* test was used; *P < .05; **P < .01

We previously found a profound activation of the IRE1α/XBP1 and ATF6 arms of the UPR in spinal cord of SALS cases leading to the expression of XBP1s and 7 of its genuine target genes (SEL1L, HERPUD1, OS9, DNAJC10, DNAJB9, PDIA4 and HSPA5).^9^ Herein, we sought to determine whether these UPR arms were activated in the motor cortex. We found that the expression of *XBP1s* and its target genes, such as *HSPA5, PDIA4* and *DNAJC10*, were increased in the motor cortex of SALS cases indicating the activation of both IRE1α/XBP1 and/or ATF6 arms (Figure 1A). Notably, the up‐regulation of these genes resembled the changes observed in spinal cord samples in terms of abundance and magnitude of the increase in the expression.^9^ At the protein level, DNAJC10 did not change in the motor cortex of SALS cases although it was strongly up‐regulated in spinal cord (Figure 1B,C), possibly due to a greater abundance in cortical tissue (Figure S1). The levels of HSPA5 and PDIA4 proteins, instead, were increased in the motor cortex and in spinal cord of SALS cases, consistent with the gene expression results (Figure 1B,C). Other ER stress target genes such as *P4HB*, *CANX* and *HYOU1* showed comparable expression between disease and healthy individuals in both spinal cord and motor cortex (Figure S1). These results indicate that ER stress occurs in the motor cortex of SALS in a similar way to spinal cord and this leads to the up‐regulation of a specific set of genes.

### 3.2 Distinctive features between the spinal cord and motor cortex derived from SALS cases in the expression of UPR genes at the mRNA and protein level

**Figure 2:**
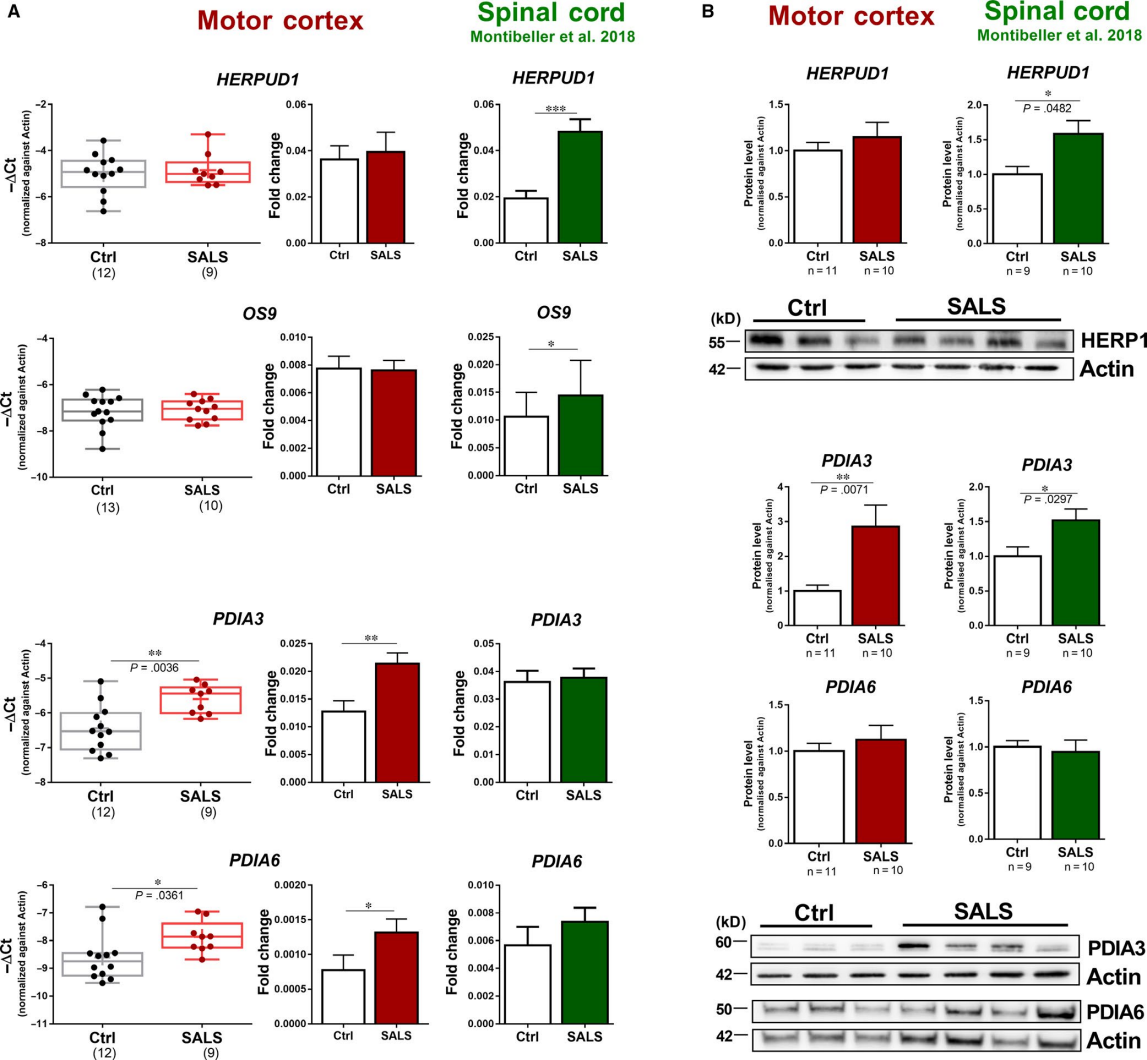
Divergent gene and protein expression in the motor cortex and spinal cord in SALS cases. A, mRNA expression analysis of HERPUD1 and OS9 in the motor cortex of healthy individuals (Ctrl, black) and sporadic cases of amyotrophic lateral sclerosis (SALS, red). B, Western blot analyses are shown for HERPUD1 detected in the motor cortex of healthy individuals (Ctrl, white) and SALS cases (SALS, red). Representative Western blots are shown for HERPUD1 in the motor cortex. C, mRNA expression analysis of PDIA3 and PDIA6 in the motor cortex of healthy individuals (Ctrl, black) and sporadic cases of amyotrophic lateral sclerosis (SALS, red). D, Western blot analyses are shown for PDIA3 and PDIA6 detected in motor cortex of healthy individuals (Ctrl, white) and SALS cases (SALS, red). Representative Western blots are shown for PDIA3 and PDIA6 in the motor cortex. Gene expression and protein expression in spinal cord samples (SALS, green) were obtained from.^8^ For gene expression, box plot and bar plot are representations for the same samples. Median, maximum and minimum values were used to represent the data as box and whiskers; mean was shown as ‘+’inside the box. Means and SEMs were used to represent the data in the bar plot. The dots represent individual samples. For protein expression, quantitative analyses of each protein were performed by densitometry of the relative bands in control and SALS cases. Values were obtained using actin as a reference gene. Means and SEMs were used to represent the data. The numbers under the graphs represent the number of samples analysed. SALS, sporadic amyotrophic lateral sclerosis; Ctrl, control. According to D’Agostino and Pearson normality test, all data are normally distributed. Unpaired *t* test was used; *P < .05; **P < .01

Although several genes showed similar expression patterns in both tissues, we found that other ER stress target genes appeared to be differentially regulated in spinal cord compared to the motor cortex in SALS cases. Indeed, genes involved in ER protein quality control (ERQC) mechanisms such as *HERPUD1* and *OS9*, which have been found to be up‐regulated in spinal cord,^9^, ^26^ showed no expression changes in the motor cortex (Figure 2A). Similarly, two other genes related to degradation processes, such as *DNAJB9* and *SEL1L*, were not differentially expressed in the motor cortex suggesting a different expression pattern between the two CNS regions (Figure S2). This was further confirmed at the protein level where HERPUD1 was increased 1.5‐fold in spinal cord of SALS cases while it did not change between control and disease cases in the motor cortex (Figure 2B). Conversely, we found that the expression of PDI family genes, such as *PDIA3* and *PDIA6*, was substantially increased only in the motor cortex (Figure 2C). While PDIA6 protein levels were slightly but not significantly increased, PDIA3 protein showed a threefold increase in the motor cortex of SALS cases consistent with the observed effect on gene expression (Figure 2C,D). PDIA3 protein was also up‐regulated in the spinal cord of SALS cases although the magnitude of the change was smaller than in the motor cortex (Figure 2D, Figure S2). No significant association was found in the expression of several representative genes between the motor cortex and the spinal cord within the same individual (Figure S3). These results suggest that ER stress pathways show a selective activation of different sets of genes in spinal cord compared to the motor cortex where PDI genes appear to be predominantly affected.

### 3.3 HSF1 target genes are differentially expressed in spinal cord, but not in the motor cortex, of SALS cases

**Figure 3:**
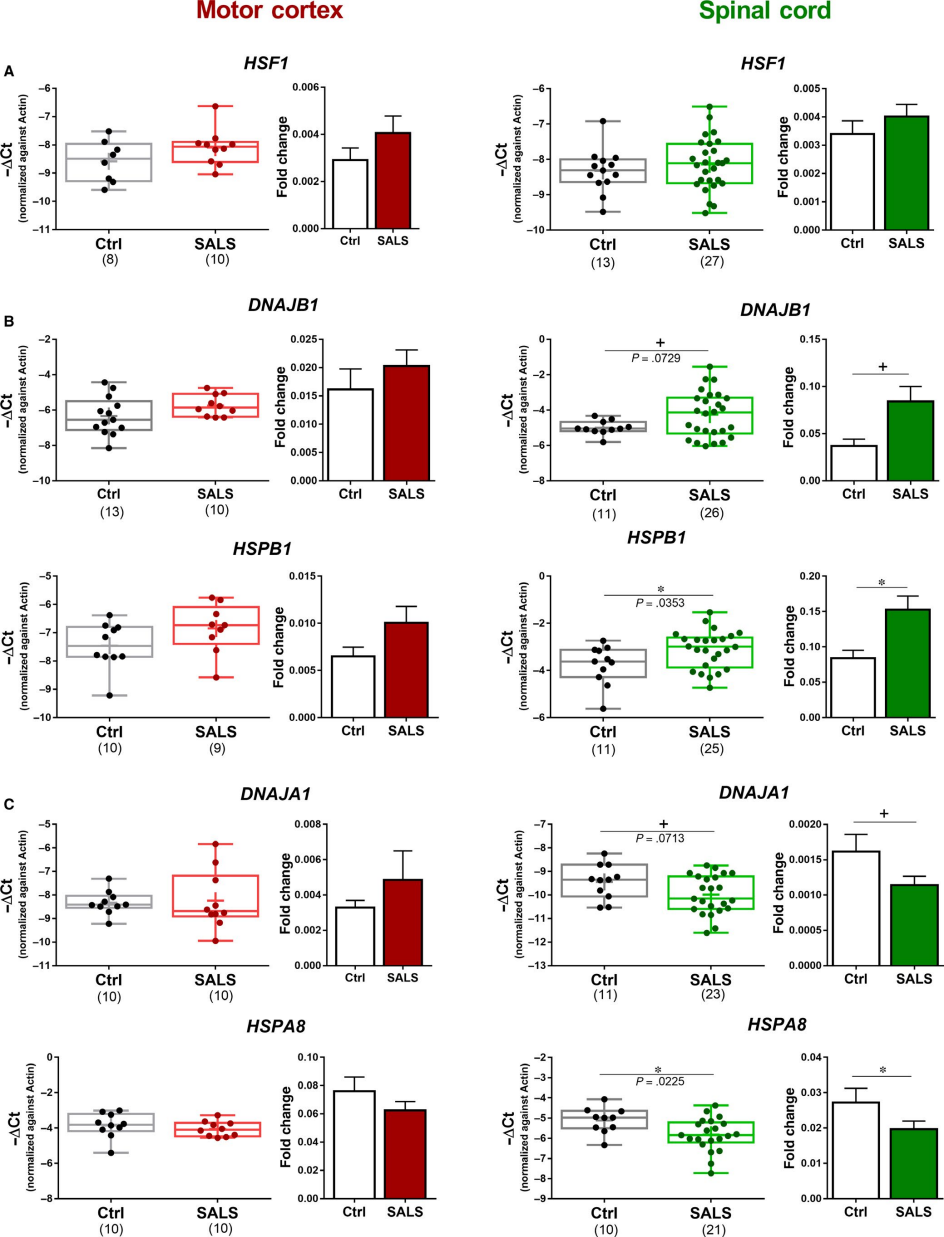
Divergent gene expression of HSF1 target genes in the motor cortex and spinal cord in SALS cases. (A) mRNA expression analysis of HSF1, (B) DNAJB1 and HSPB1, (C) DNAJA1 and HSPA8 in the motor cortex (red) and spinal cord (green) of healthy individuals (Ctrl, black) and sporadic cases of amyotrophic lateral sclerosis (SALS). Box plot and bar plot are representations for the same samples. Median, maximum and minimum values were used to represent the data as box and whiskers; mean was shown as ‘+’inside the box. Means and SEMs were used to represent the data in the bar plot. The dots represent individual samples, and the numbers under the graphs represent the number of samples analysed. SALS, sporadic amyotrophic lateral sclerosis; Ctrl, control. According to D’Agostino and Pearson normality test, all data are normally distributed. Unpaired *t* test was used; +: 0.1 < *P* > .05, *P < .05; **P < .01

Subsequently, we investigated whether the activation of another cellular stress response, the heat shock response (HSR), was differentially regulated in the spinal cord and motor cortex of SALS cases. As previously described,^16^ we found that one of the major HSF1 target gene, *HSPB1,* was up‐regulated in spinal cord of SALS cases. Moreover, *HSF1* and its target *DNAJB1* showed a trend to increased expression (Figure 3A,B) suggesting that the HSF1 pathway was activated in spinal cord of SALS cases. Surprisingly, the expression of the other two HSF1 target genes, *HSPA8* and *DNAJA1*,^27^ was decreased (Figure 3C) in this tissue. HSPA8 and DNAJA1 belong to a ‘C3HC4‐type RING finger domain binding’ Gene ontology (GO) term.^28^ Interestingly, all members of this cluster (HSPA8, DNAJA1, KCNH2, PINK1, HSPA1A and HSPA1B) were down‐regulated in lumbar spinal cord of SALS cases^29^ (Table S3). In this regards, other HSF1 target genes such as HSP40 and HSP70 have also been found to be down‐regulated^14^ suggesting a more complex regulation of this pathway in SALS spinal cord. In contrast, none of the analysed HSF1 target genes showed any change in expression in the motor cortex of SALS cases (Figure 3A‐C). These results suggest that, while the HSR is activated in spinal cord, it appears to remain latent in the motor cortex.

### 3.4 Analysis of UPR gene expression in frontal and temporal cortex of FTLD cases reveals tissue‐selective patterns

**Figure 4:**
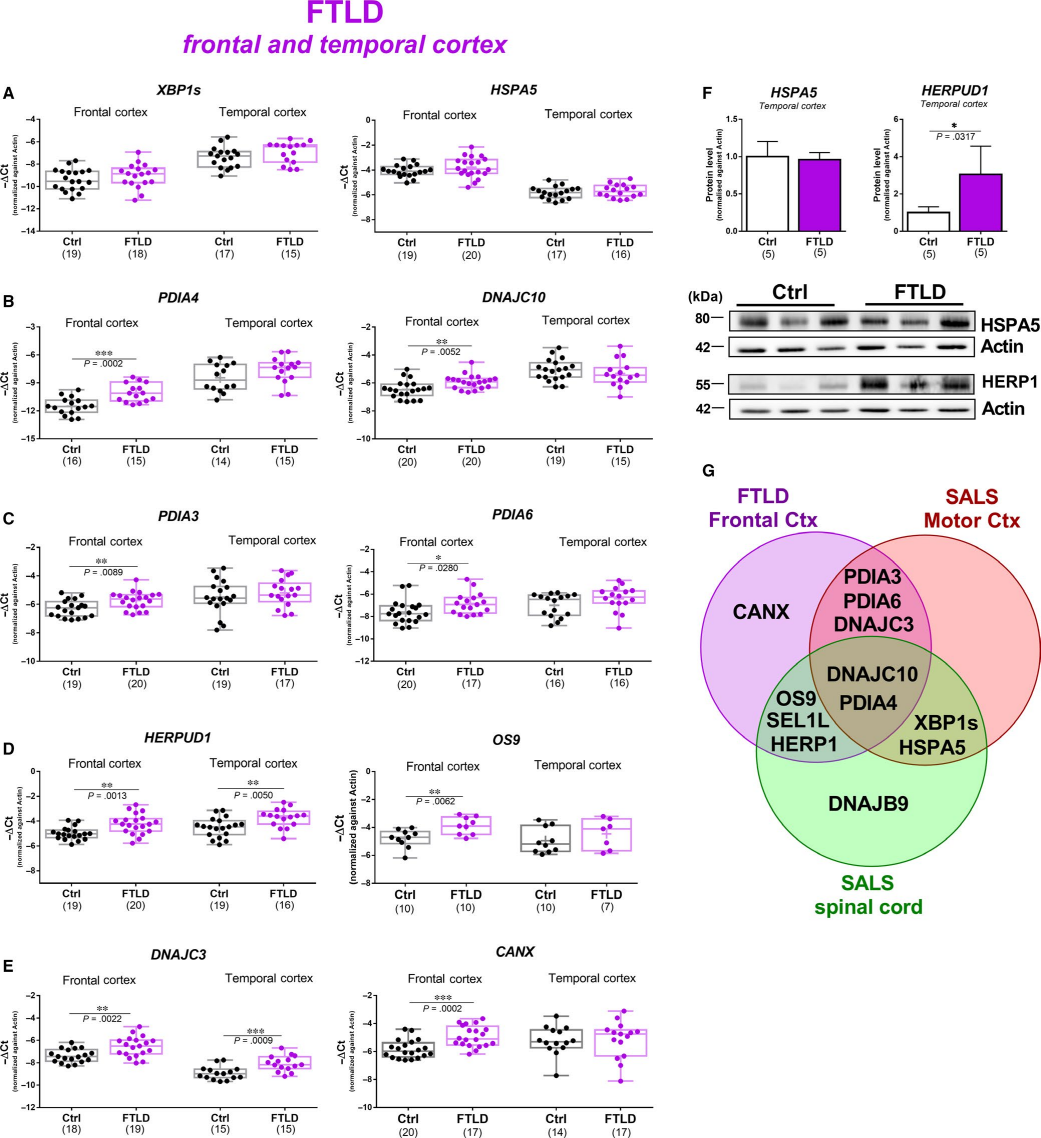
Gene and protein expression of ER stress genes in cortical regions derived from FTLD cases. mRNA expression analysis of (A) XBP1s and HSPA5, (B) PDIA4 and DNAJC10, (C) PDIA3 and PDIA6, (D) HERPUD1 and OS9 and (E) DNAJC3 and CANX in frontal and temporal cortex of healthy individuals (Ctrl, black) and frontotemporal lobar degeneration cases (FTLD, orange). Box plot and bar plot representations for the same samples. Median, maximum and minimum values were used to represent the data as box and whiskers plots; mean was shown as ‘+’inside the box. Means and SEMs were used to represent the data in the bar plot. The dots represent individual samples. (F) Western blot analyses are shown for HSPA5 and HERPUD1 detected in the temporal cortex of healthy individuals (Ctrl, white) and FTLD cases (FTLD, orange). Quantitative analyses of each protein were performed by densitometry of the relative bands in control and SALS cases. Values were obtained using actin as a reference gene. Means and SEMs were used to represent the data. Representative Western blots are shown for HSPA5 and HERPUD1 in the temporal cortex. The numbers under the graphs represent the number of samples analysed. FTLD, frontotemporal lobar degeneration; Ctrl, control. According to D’Agostino and Pearson normality test, all data are normally distributed. Unpaired *t* test was used; *P < .05; **P < .01; ***P < .001. (G) Venn diagram for the upregulated genes found in the motor cortex and spinal cord of SALS cases and frontal cortex of FTLD cases

As HSR genes were not significantly activated in the motor cortex, we focused on the UPR. We tested whether the differences between the spinal cord and the motor cortex were mainly due to a tissue or disease specificity. To this end, we investigated the expression of UPR genes in two cortical regions, frontal and temporal cortex, derived from frontotemporal lobar degeneration (FTLD) cases. Frontotemporal lobar degeneration is a common form of dementia which exhibits a significant clinical, neuropathological and genetic overlap with ALS.^1^ The FTLD cases were all neuropathologically characterized by TDP‐43^+ve^ inclusions and lacked hexanucleotide repeat expansions in *C9ORF72.* These cases showed no changes in the expression of *XBP1s* and *HSPA5* genes in both cortical regions, suggesting that the IRE1α/XBP1 arm was not activated (Figure 4A). However, the activation of ER stress responses was confirmed by the up‐regulation of several UPR target genes such as *PDIA4* and *DNAJC10* in frontal cortex of FTLD cases (Figure 4B). We found that *PDIA3* and *PDIA6* were also up‐regulated in FTLD cases (Figure 4C) showing the prominent up‐regulation of *PDIA3* and the more modest up‐regulation of *PDIA6* observed in the motor cortex of SALS cases (Figure 2C). ER protein quality control genes such as *HERPUD1*, *OS9* and *SEL1L,* which were found to be up‐regulated in SALS spinal cord, were also increased in frontal cortex of FTLD (Figure 4D, Figure S4). Interestingly, *CANX* was the only gene that was up‐regulated exclusively in the frontal cortex of FTLD cases (Figure 4D). Notably, expression of *CANX* was found to be increased in the same CNS region of Alzheimer's disease cases suggesting a specific role of this gene in frontal cortex.^9^ Notably, although most of the changes were detected in frontal cortex, *HERPUD1* and *DNAJC3* were also significantly up‐regulated in temporal cortex of FTLD cases (Figure 4D, Figure S4). Gene expression results were confirmed by Western blot which showed no changes in the expression of HSPA5 but a substantial increase in HERPUD1 protein in temporal cortex of FTLD cases (Figure 4F). Analysis of mRNA expression changes in FTLD frontal cortex SALS, spinal cord and motor cortex unveiled that the expression of *PDIA6* and *DNAJC3* was increased mainly in cortical regions while the *DNAJB9* gene was up‐regulated only in the spinal cord (Figure 4G). Similarly, *PDIA3*mRNA was found up‐regulated predominately in cortical regions and its expression was also increased in spinal tissue at the protein level. A disease specificity was suggested by the SALS‐selective overexpression of XBP1s and HSPA5 (Figure 4G). Finally, the expression of *DNAJC10* and *PDIA4*mRNA was increased in both disorders across all tissues.

### 3.5 CNS cell type markers correlate with specific HSR and UPR genes in motor cortex and spinal cord of SALS cases

**Figure 5:**
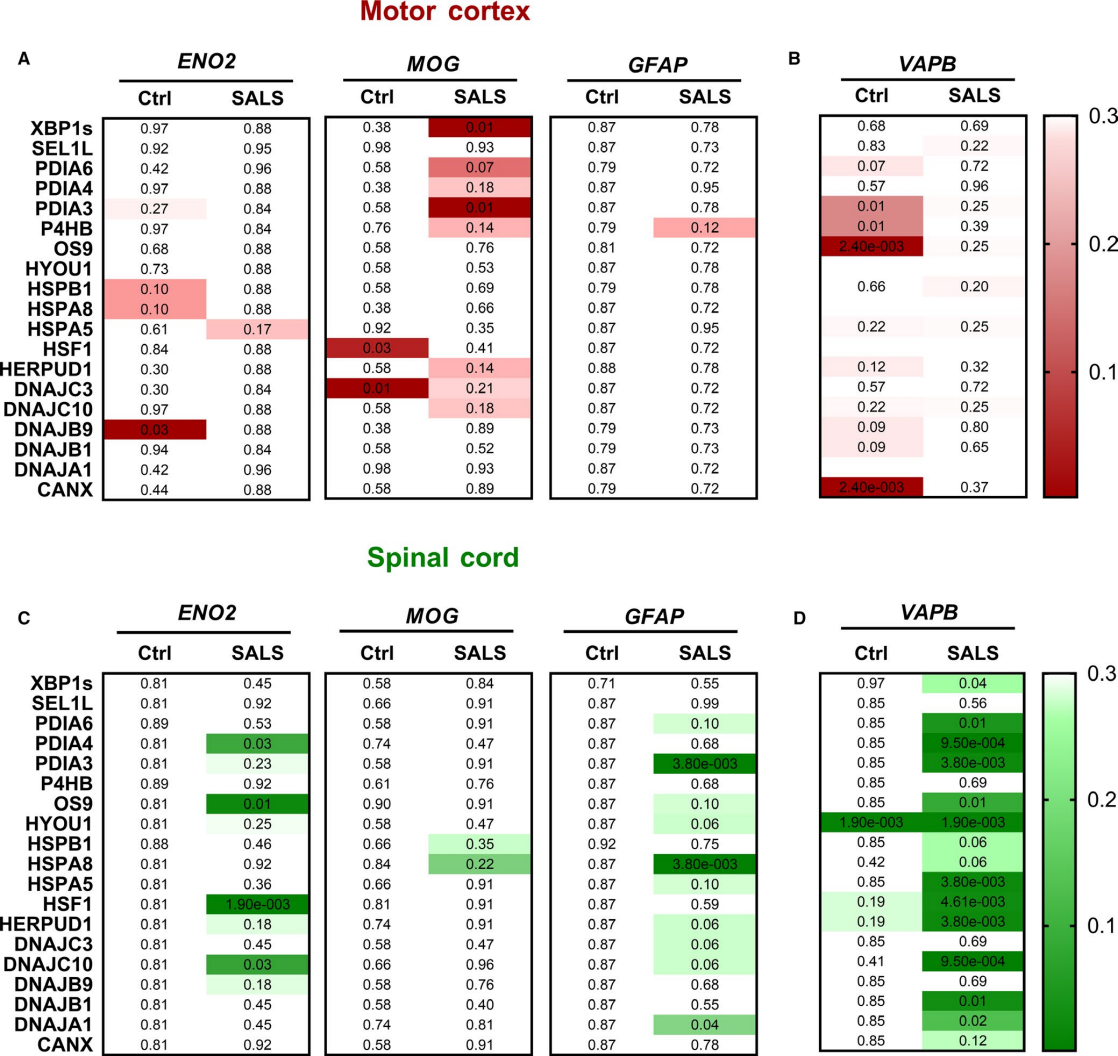
Correlation analyses between cell type markers and UPR‐HSR genes in the motor cortex and spinal cord. A‐D, Heat map of the FDR‐corrected *P* values derived from correlation analyses between UPR‐HSR and cell type–specific marker genes in the motor cortex (red) and spinal cord (green). Cell type–specific markers used were as follows: ENO2 for neurons, MOG for oligodendrocytes, GFAP for astrocytes and VAPB for motor neurons. Each box of the heat maps, divided into two columns (Ctrl, SALS), corresponds to a cell type marker analysed, and each raw corresponds to a specific gene. The Pearson correlation's test was used when data were normally distributed (according to D’Agostino and Pearson normality test); the Spearman correlation's test was used only for SEL1L because the data were not sampled from a Gaussian distribution. The Benjamini‐Hochberg FDR test was used to correct *P* values for multiple testing. Ctrl, control; SALS, sporadic amyotrophic lateral sclerosis. Cell values represent the FDR‐corrected *P* value. The coloured bar indicates the range of intensity values for each gene in the heat maps

To understand which cell populations are responsible for the expression of UPR and HSR target genes, we investigated the correlation between these genes and cell type–specific markers. To this end, we selected three well‐characterized cell type markers for the three major CNS cell types: GFAP for astrocytes,^17^, ^18^, ^23^, ^24^ MOG for oligodendrocytes^19^, ^20^, ^23^, ^24^ and ENO2 for neurons.^21^, ^22^, ^25^, ^30^ In the motor cortex, the expression of all three markers was comparable between SALS and control cases (Figure S5). The neuronal marker ENO2 showed an FDR‐corrected significant correlation with DNAJB9 in healthy individuals but not in SALS cases (Figure 5A). Correlation analyses with MOG revealed a robust association between this oligodendrocyte marker and UPR genes in the motor cortex. HSF1 and DNAJC3 correlated with MOG in control cases while XBP1s and PDIA3 showed a strong correlation in SALS cases (Figure 5A). Conversely, none of the analysed genes showed a correlation with GFAP (Figure 5A). In spinal cord, the expression of oligodendrocyte and astrocyte markers was similar between control and SALS cases while the neuronal marker was down‐regulated in disease cases (Figure S5) indicating the loss of a neuronal subpopulation associated with ALS (eg motor neurons). Although none of the genes correlated with *ENO2* in healthy cases, we found that *PDIA4*, *OS9*, *HSF1* and *DNAJC10* showed a strong correlation with the neuronal marker in spinal cord of SALS cases (Figure 5B). It is important to note that, while several genes were up‐regulated, *ENO2* was down‐regulated in SALS cases indicating that the correlation analyses are not primarily affected by gene expression changes. None of the stress genes correlated with *MOG* in spinal cord of control and disease cases (Figure 5B), while the astrocytic marker correlated negatively with *HSPA8*, *PDIA3* and *DNAJA1* (Figure 5B, Figure S6). These results indicate that UPR target genes strongly correlated with the oligodendrocyte marker in motor cortex, whereas a different group of UPR and HSR target genes correlated with the neuronal marker in spinal cord of SALS cases.

### 3.6 VAPB is a motor neuron marker and correlates with several ER stress genes in spinal cord of SALS cases

To further investigate the specificity of the gene expression changes, we investigated the correlation between ER stress genes and a marker for MNs, the main neuronal subpopulation affected in ALS.^1^ VAPB has been found to be highly expressed in spinal cord compared to any other tissues in the human body^31^ and enriched in motor neurons both at the mRNA and protein level where it is involved in several functions.^16^, ^32^, ^33^ Moreover, pathogenic mutations in *VAPB* gene, which are associated with ALS, promote the formation of ubiquitinated aggregates and lead to the generation of a robust motor phenotype in vivo.^32^ Based on this evidence, we have been suggested that VAPB may be considered as a new putative motor neuron marker. Accordingly, VAPB showed a poor correlation with oligodendrocyte and astrocyte markers, while it was strongly correlated with the neuronal marker, *ENO2,* in both motor cortex and spinal cord (Figure S8). Next, in a previous series of experiments where spinal cord sections were immunostained for VAPB and quantified together with the number of large motor neurons.^34^ VAPB expression correlated positively with the number of motor neurons (Figure S8). Interestingly, the VAPB data distribution enabled a better separation between SALS and control cases compared to ChAT, a well‐established marker for MNs^29^ (Figure S8). Furthermore, we analysed the correlation between VAPB and MNX1, also known as HB9, which has been demonstrated to be one of the most reliable marker for mature MNs.^35^, ^36^ MNX1 showed a remarkable correlation with VAPB (*P* = .001, *r* = .7526), and its expression was decreased in the SALS spinal cord similarly to VAPB (Figure S5 and S8). These evidences showed that VAPB could be used as a reliable marker for motor neurons. Consequently, we performed a correlation analyses between stress genes and the new putative motor neuron marker. In motor cortex of control cases, the *VAPB* expression strongly correlated with the expression of *OS9*, *CANX, PDIA3* and *P4HB* (Figure 5C). Interestingly, genes that correlated with the neuronal marker *ENO2,* such as *DNAJC10*, *PDIA4, OS9* and *HSF1,* showed an even stronger correlation with *VAPB* in spinal cord of SALS cases (Figure 5D). Similarly, other genes such as *HERPUD1*, *PDIA3* and *HYOU1* showed a remarkable correlation with the motor neuron marker (Figure 5D). *HYOU1* was the only gene that showed a correlation with *VAPB* in both healthy and disease cases (Figure 5D). Moreover, *XBP1s* and its targets (*HSPA5* and *PDIA6)* together with *HSF1* and its targets (*DNAJB1* and *DNAJA1)* also correlated with the MN marker in SALS cases (Figure 5D) indicating the involvement of these pathways in motor neurons in disease conditions.

### 3.7 Cellular localization of induction of UPR target genes

**Figure 6:**
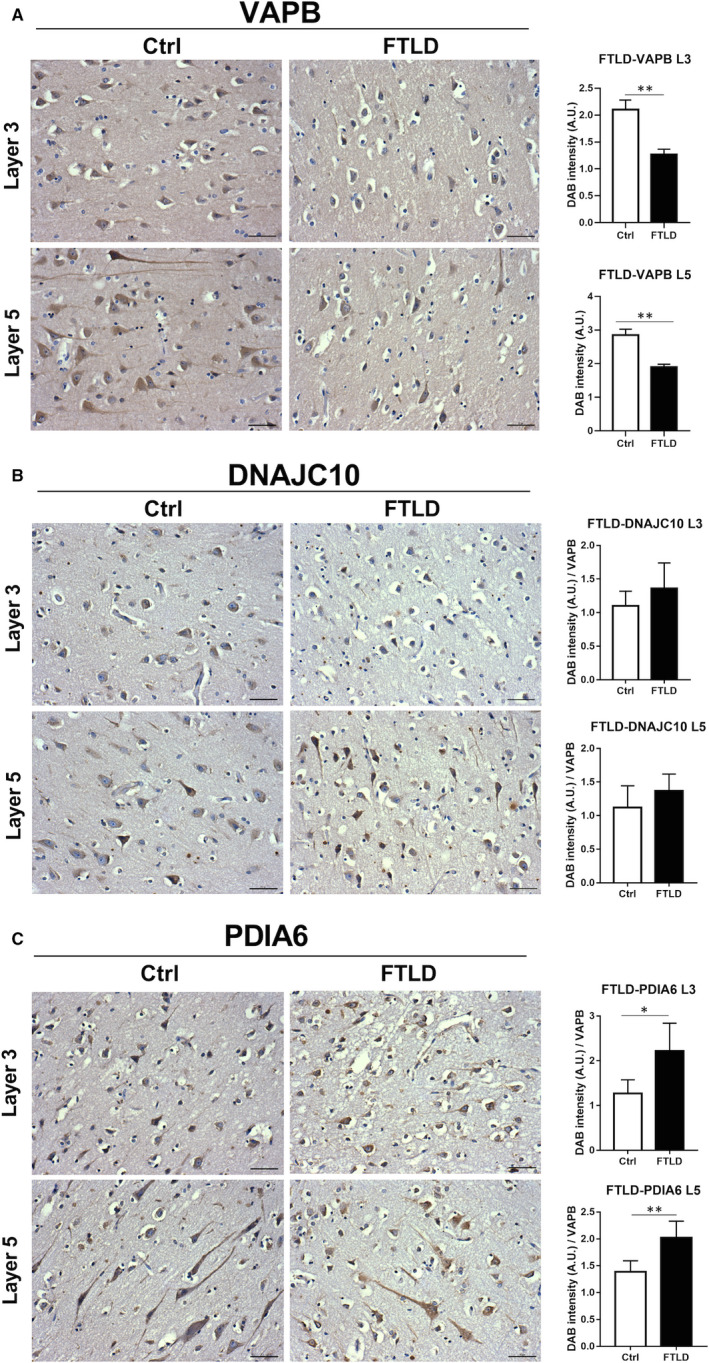
Immunohistochemical localization of VAPB, DNAJC10 and PDIA6 in frontal cortex from FTLD cases and controls. A, Representative immunohistochemically stained frontal cortex from FTLD cases and controls showing cellular localization of VABP staining in cortical layers 3 (L‐3) and 5 (L‐5). Strong neuronal staining for VABP is seen in large pyramidal neuronal cell bodies and their axons, which is particularly prominent in L‐5. All images at X20 magnification. Scale bar = 50 μm. In FTLD cases, there is substantial atrophy and shrinking of neurons. Histograms showing quantification of VAPB staining of FTLD cases and controls in frontal cortex from L3 and L5. Two‐tailed unpaired *t* test was used. *P < .05, **P < .01. n = 5. B and C, Representative immunohistochemically stained frontal cortex from FTLD cases and controls showing cellular localization of (B) DNAJC10 and (C) PIA6 staining in cortical layers 3 (L‐3) and 5 (L‐5). Moderate to strong neuronal staining for DNAJC10 and PDIA6 is seen in large pyramidal neuronal cell bodies and their axons, which is particularly prominent in L‐5. All images at ×20 magnification. Scale bar = 50 μm. In FTLD cases, DNAJC10 staining remains strong but this accompanied by atrophy and shrinking of neurons. PDIA6 staining is strong in cell bodies and their axons in controls while these cells are diminished in FTLD and accompanied by atrophied cells. Histograms showing quantification of DNAJC10 and PDIA6 staining relative to VAPB staining of FTLD cases and controls in frontal cortex from L3 and L5. Two‐tailed unpaired *t* test was used, n = 5. Two‐tailed unpaired *t* test was used. *P < .05, **P < .01. n = 5[Correction Statement: Correction added on xx April 2020 after first online publication: Figure 6 has been updated in this version.]

**Figure 7:**
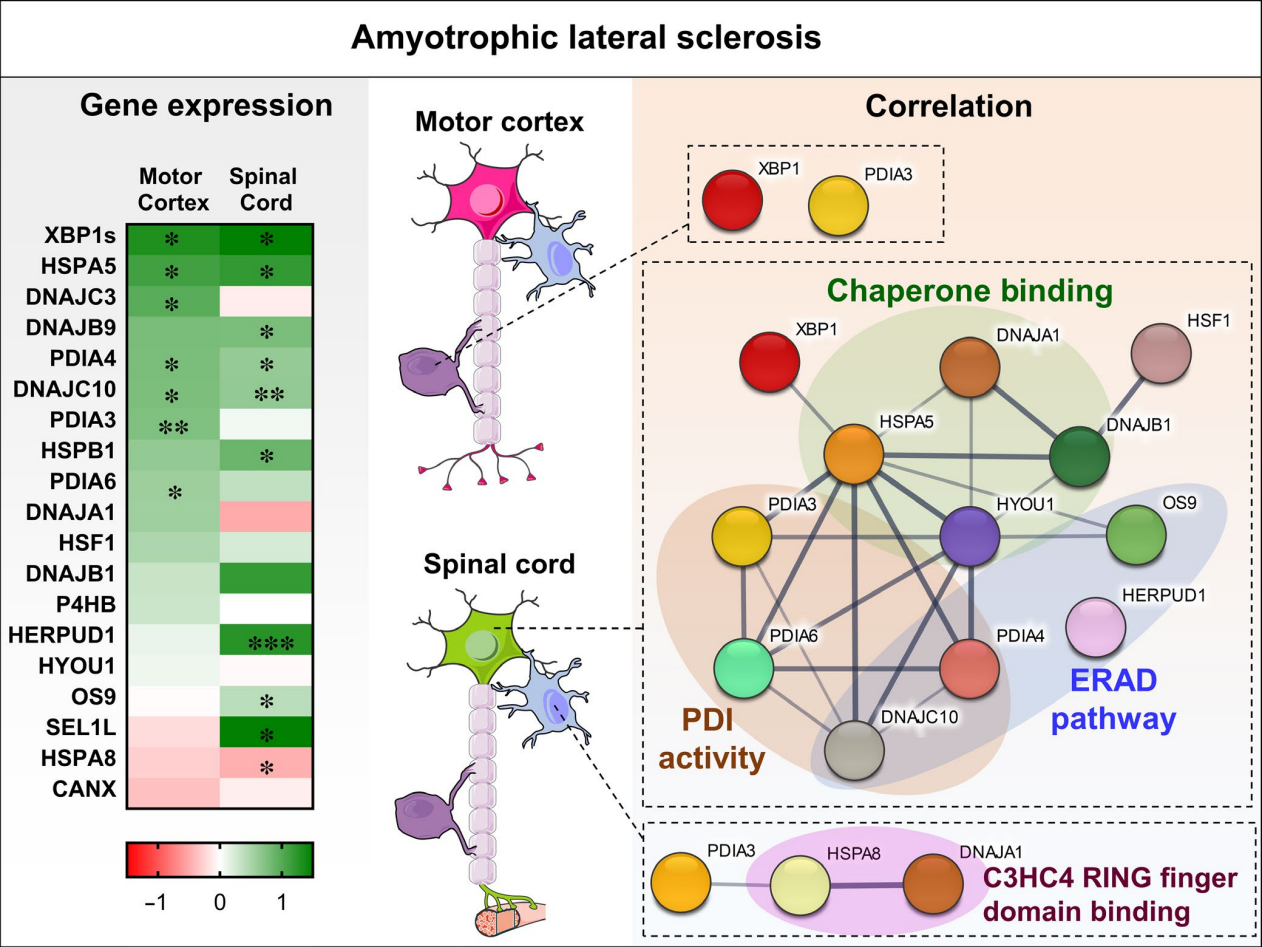
Comparison between gene expression profiles and protein‐protein interaction analysis in the spinal cord and motor cortex. (left) Heat map of the gene expression of the UPR and HSR genes analysed in the spinal cord and motor cortex of SALS cases. The gene expression was normalized against the controls, and log_2_ FC was plotted. Each column in the heat maps corresponds to a tissue analysed, and each row corresponds to a specific gene. The coloured bar indicates the range of intensity values for each gene in the heat maps. Stars represent the genes that were changing significantly. *P < .05; **P < .01; ***P < .001. (right) Protein‐protein interaction network generated by STRING 11.0 (Search Tool for the Retrieval of Interacting Genes/Proteins) database. The connecting lines indicate functional relationships and direct protein‐protein interactions. Line thickness indicates the strength of data support. Different colours reflect different proteins. Genes were grouped by their main function

Initially, Western blot analyses were carried out to confirm the up‐regulation ER stress genes and support the mRNA changes for HSPA5, PDIA4 and PDIA3 which were significantly up‐regulated in SALS motor cortex and for HSPA5, DNAJC10, HERPUD1, PDIA3, DNAJB1 and HSPB1 that were all significantly up‐regulated in SALS spinal cord. Similarly, HERPUD1 protein was significantly up‐regulated in FTLD cases in the temporal cortex. We then carried out immunohistochemistry of DNAJC10, PDIA6 and VAPB to localize the cellular distribution of these protein in control tissue (Figure 6A‐E). All three proteins showed high to medium levels of staining in large pyramidal neurons in layers 3 and 5 of frontal cortex with low to moderate staining in glial cells (Human Protein Atlas).^37^ Levels of DNAJC10 and PDIA6 were very low in the neuropil. In FTLD cases, extensive neurodegenerative changes were evident characteristic of this condition, namely atrophy and shrinkage neurons, vacuolization and neuronal loss (Figure 6A‐E). As shown at protein level, this is accompanied by a loss in VAPB protein and at the cellular level by a significant loss in overall VAPB immunostaining (Figure 6A,B). To determine whether the mRNA changes occurring in UPR associated genes in disease were accompanied by significant changes in protein staining, the staining of each protein significantly induced in FTLD, DNAJC10 and PDIA6 were related to VAPB staining in layers 3 and 5 of frontal cortex, which reflects neuronal loss. This approach avoids the need to identify specific cell types in disease which is complex due to their heterogeneity (eg the atrophy, shrinking and vacuolization that occurs in pyramidal neurons). No significant changes were found for DNAJC10 (Figure 6C,D) but substantial significant up‐regulation of PDIA6 staining was found in both cortical layers studied (Figure 6C). These findings help to substantiate the findings for PDIA6 that are associated with a neuronal localization. Moreover, the analysis of protein‐protein interactions (PPI) from STRING repository showed a physical interaction between PDIA6 and DNAJC10, which, together with other molecules involved in proteostasis, form an interconnected network (Figure 7) strongly associated with motor neurons during disease progression.

## 4 DISCUSSION

The distinctive feature of ALS is the degeneration of motor neurons in both motor cortex and spinal cord.^38^, ^39^ However, the interaction and the cellular mechanisms shared between these two tissues remains poorly characterized. Herein, we investigated the activation status of two stress pathways related to the control of protein homeostasis, the UPR and the HSR, in both motor cortex and spinal cord of SALS cases.

### 4.1 Dominance of Protein disulphide isomerase activity vs. protein quality control in cortical regions

The up‐regulation of *XBP1s* and its downstream targets, such as *HSPA5*, indicates that the UPR is activated similarly in motor cortex and spinal cord of SALS cases.^9^ However, the IRE1/XBP1 pathway activation led to increased expression of a subset genes involved predominately in ER protein quality control in spinal cord, while up‐regulation of PDIs such as *PDIA3*, *PDIA4*, *DNAJC10* (also known as *PDIA19*) and *PDIA6* was observed in motor cortex. Notably, several ALS‐causative mutations have been found in PDIs genes highlighting their functional role in this disorder.^40^, ^41^ Therefore, the overexpression of PDIs proteins observed in ALS may reflect a cellular stress response to cope with altered protein homeostasis and suggest that modulation of PDI function/activity could have a more therapeutically beneficial outcome when targeted in the motor cortex compared to spinal cord.^41^ In SALS spinal cord, instead, we found a substantial activation of HSR through the up‐regulation of *HSPB1* and *DNAJB1* and the down‐regulation of *HSPA8* and *DNAJA1*. Interestingly, the last two genes belong to the ‘C3HC4‐type RING finger domain binding’ GO term (Figure 7), described to be essential for the E3 ligase and ubiquitin‐dependent processes,^42^ found to be impaired in ALS.^43^ On the other hand, in agreement with previous observations,^14^, ^44^ we found no evidence of HSR activation in SALS motor cortex probably due to the transient nature of this process, which could not be detectable in post‐mortem tissue.^45^ Thus, while the activation of UPR and HSR promoted the expression of ERQC genes in spinal cord, UPR, but not HSR, predominately induced the expression of PDIs in the motor cortex of ALS cases, most likely to control protein redox mechanisms.

### 4.2 Identifying the cell types subjected to induction of ER stress target genes

To identify the major cell types involved, we correlated the expression of the UPR and HSR genes with cell type‐specific markers such as *GFAP* for astrocytes, *MOG* for oligodendrocytes and *ENO2* for the neuronal population (Figure 6B,C). We found that increased expression of select ER stress genes such as *DNAJC3* strongly correlated with the oligodendrocyte marker in the motor cortex of healthy individuals while *XBP1s*, *PDIA3, DNAJC10*, *PDIA4* and *PDIA6* correlated or showed a positive correlation trend with MOG in SALS cases (Figure S5). Interestingly, an increase in PDI expression, preceded by the activation of both IRE1/XBP1 and ATF6 pathways, has been reported to occur during active myelination in cortical regions of rats.^46^ In line with this, a strong PDI expression in oligodendrocytes was observed in SOD1 mice,^47^ which was characterized by abnormalities in plasma and membrane lipid signalling, especially in the early symptomatic stages of ALS.^47^ In spinal cord, specific PDIs including *PDIA4* and *DNAJC10* correlated with *ENO2*, whose expression was decreased. This down‐regulation could reflect the neuronal loss, which was more evident in spinal cord probably due to a lower heterogeneity in neuronal cell composition compared to the motor cortex.^48^, ^49^ Together with these, other genes involved in degradation mechanism such as *OS9* showed a strong correlation with *ENO2* in SALS compared to the control groups suggesting that the expression alteration of these genes might occur specifically in neurons of disease cases.^50^, ^51^, ^52^ Whereas none of the ER stress genes correlated with MOG, three genes (*PDIA3*, *HSPA8* and *DNAJA1*) showed a negative correlation with GFAP suggesting an inverted relationship between astrocytes and UPR response. These results indicate that UPR genes, in particular PDIs, correlate with the oligodendrocyte marker in the motor cortex while other genes, involved predominately in ERAD, correlate with the neuronal marker in spinal cord of SALS cases.

To further document the neuronal cell types harbouring each UPR and HSR response, we investigated the correlation between these genes and a motor neuron marker. VAPB, an ER transmembrane protein involved in the regulation of vesicle trafficking, showed a strong correlation with the neuronal marker ENO2 as well as MNX1, a marker for mature motor neurons.^35^, ^36^ In addition, we demonstrated that VAPB localized to large motor neurons in human spinal cord^34^ dividing ALS cases from healthy control even better than the well‐established motor neuron marker ChAT. In the motor cortex, we found that VAPB correlated with *CANX*, *OS9*, *PDIA3* and *P4HB* in control cases. Interestingly, mutations in *PDIA3* and *P4HB* genes have been related to motor neuron dysfunction.^40^, ^41^ OS9, instead, has been demonstrated to be involved in the degradation of hypoxia‐inducible factor 1alpha,^53^ a key factor in the hypoxic stress sensor pathway which is impaired in motor neurons of ALS mouse models.^54^ It is important to note that none of the ER stress genes correlated with a motor neuron marker in SALS cases in the motor cortex. Conversely, almost all the UPR and HSR genes showed a strong correlation with VAPB in SALS spinal cord. In agreement with these results, ER stress pathways have been demonstrated to be activated in iPSC‐derived motor neurons, obtained from patients affected by ALS^55^ and in motor neurons of SOD1 transgenic mice from the early stages of ALS pathogenesis.^8^, ^47^ The expression of *DNAJC10*, *HSPA5*, *PDIA6*, *PDIA4*, *DNAJA1* and *DNAJB1* strongly correlated to ALS compared to control group indicating that these genes are somehow linked to the disease (Figure S8). Moreover, we found that DNAJC10 and PDIA6 proteins, together with VAPB, showed moderate to high intensity in large pyramidal neurons supporting the correlation analyses obtained from gene expression data. These results also suggest a role of VAPB in ERQC process and UPR modulation, as previously reported.^32^, ^56^ In fact, it has been demonstrated that ALS‐associated mutations in VAPB blunts the activation of IRE1α/XBP1 32, 33 as well as that of ATF6^57^ thereby affecting protein folding and triggering the formation of intracellular aggregates.^58^ Although the specific molecular mechanism needs to be fully characterized, the modulation of the interaction between VAPB and IRE1α/ATF6 may represent an interesting therapeutic approach in order to re‐establish the intracellular protein homeostasis in ALS targeting specifically spinal motor neurons. Finally, protein‐protein interaction analyses revealed that several genes associated with spinal motor neurons were part of an interconnected network (Figure 7) from which we could identify three key functional clusters: chaperones binding, PDI activity and the ERAD pathway. In agreement, several studies have demonstrated the role of protein quality control in spinal motor neurons.^35^, ^36^, ^55^ Moreover, in motor cortex, the oligodendrocyte‐associated signalling network comprising XBP1‐PDIA3 is supported by evidences that showed that gene therapy employed to deliver active XBP1 (XBP1s) into ALS mouse models had a significant impact on motor recovery after spinal cord injury, which was associated with enhanced oligodendrocyte survival.^59^

These results have demonstrated for the first time in human post‐mortem tissue that ER stress signalling is strongly activated in the motor cortex and spinal cord of SALS cases. In addition, correlation analyses revealed that specific subsets of these genes are associated with specific CNS tissue and specific cell type supporting the idea that these stress pathways might have a broad impact on non‐cell autonomous aspects of ALS. The identification of the key players of these pathways provides a valuable source of information to develop new tissue and cell type selective therapeutic strategies in ALS.

## CONFLICT OF INTEREST

The authors declare no conflict of interest.

## AUTHORS' CONTRIBUTIONS

LM and JB designed research; LM, TLY and KKJ performed research; PP contributed with supplementary data; JB contributed reagents/analytical tools; LM analysed data; and LM and JB wrote the manuscript. All authors read and approved the final manuscript.

## ETHICAL APPROVAL

The study was approved by the Riverside Research Ethics Committee. Consented tissue donors were recruited by the Motor Neurone Disease Association from throughout the UK to the Imperial College ALS Research Group (currently located on the Hammersmith Hospital campus). The initial ethical approval was obtained from the Riverside Ethical Committee and renewed as appropriate. NRES Committee Yorkshire & The Humber—Leeds Central Yorkshire and Humber REC Office (Study title: Gene expression in Motor Neurone Disease/ Amyotrophic Lateral Sclerosis (ALS), REC reference: 12/YH/0282.

## Supporting information

Supplementary Material

## Data Availability

The data that support the findings of this study are available on request from the corresponding author. The data are not publicly available due to privacy or ethical restrictions.
