# Supplementary material for: Tissue‐selective regulation of protein homeostasis and unfolded protein response signalling in sporadic ALS
Source: J Cell Mol Med. 2020 Apr 23;24(11):6055–69. doi: 10.1111/jcmm.15170 (PMC7294118; doi:10.1111/jcmm.15170)
Supplement: Supplementary file 1 — Supplementary Material [file JCMM-24-6055-s001.docx]

**Supporting Information**

***SI Material and Methods***

***Tissue samples***

The study was approved by the Riverside Research Ethics Committee. Consented tissue donors were recruited by the Motor Neurone Disease Association from throughout the UK to the Imperial College ALS Research Group (currently located on the Hammersmith Hospital campus). All ALS patients were diagnosed according to the El Escorial criteria which requires the presence of both upper motor neuron and lower motor neuron symptoms [1, 2] . The initial ethical approval was obtained from the Riverside Ethical Committee and renewed as appropriate. NRES Committee Yorkshire & The Humber - Leeds Central Yorkshire and Humber REC Office (Study title: Gene expression in Motor Neurone Disease/ Amyotrophic Lateral Sclerosis (ALS), REC reference: 12/YH/0282.

***Subjects***

Frozen motor cortex tissue was obtained from 23 cases. Controls had no neuropathological signs aside from age-related changes. Gender, age at death and post-mortem delay (PMD) of subjects are listed in Table S1. Frozen frontal cortex and temporal cortex (TCtx) tissue was obtained from 20 cases each of frontotemporal lobar degeneration (FTLD) and controls. Controls had no neuropathological signs aside from age-related changes, FTLD cases were identified by the presence of TDP43-positive inclusions. FTLD cases were genetically screened for C9orf72 mutations and C9orf72 mutation-negative were considered for this study. Sex, age at death, post-mortem delay, and clinical details of the patient samples are reported in Montibeller *et al.* [1] . There was no significant difference between groups and controls, except for an earlier age at death in the FTLD group.

***Human tissue samples preparation***

Dissected brain tissue was snap frozen, then stored at -80°C until further use. For ALS cases, frozen motor cortex tissue sampled at levels BA4 was used. For the spinal cord cases, frozen lumbar tissue sampled at levels L3 to L5 was used. For frozen frontal cortex and temporal cortex tissue, all clinical diagnoses were confirmed neuropathologically at post-mortem. Dissected brain tissue was snap frozen, then stored at -80°C until further use. Frozen frontal cortex and temporal cortex tissue were obtained from the MRC London Neurodegenerative Diseases Brain Bank, a member of the Brains for Dementia Research Network. All tissue was obtained from voluntary donors in compliance with the Mental Capacity Act (2005), and the Brain Bank has been approved by the National Research Ethics Service.

***RNA extraction and cDNA synthesis***

mRNA from frozen tissue samples and stored at -80°C. RNA was extracted from motor cortex, spinal cord, frontal and temporal cortex samples following Direct-zol RNA mini prep (Zymo Research) protocol. RNA purity and integrity for all tissue samples was assessed as reported in Montibeller *et al.* [1] .. For motor cortex tissue A260/280 absorbance ratios in all mRNA extractions and were typically in the range of 1.91 to 2.08 (Mean=1.89), indicating a high level of purity. The RNA obtained was reverse transcribed to cDNA using cloned an Avian Myeloblastosis Virus (AMV) cDNA synthesis kit (Invitrogen). Primer3 web software was used to design primers for the amplification of target cDNA sequence. In addition, KiCqStart^®^ SYBR^®^ Green Primers (Sigma) were used. To optimize the temperature gradient PCR was performed to optimise conditions for each primer and the PCR products were checked on agarose gels. Primers are listed in Table S2.

***Selection of cell type markers***

The specificity of ENO2, MOG and GFAP as cell type specific markers was confirmed by reviewing single-cell studies [3–5] . Specifically, ENO2 was associated with excitatory neurons and absent in inhibitory neurons, all oligodendrocytes & OPCs and astrocytes localised in cerebellar hemisphere [5] . In the same study, MOG and GFAP were detected only in all oligodendrocytes & OPCs and cerebellar hemisphere astrocytes, respectively. In addition, MOG and GFAP specificity for oligodendrocytes and astrocytes was confirmed by Habib *et al.* and Spaethling *et al.* [3, 4] . We selected VAPB as a putative motor neuron marker since it was detected exclusively in lumbar spinal cord motor neurons [2, 6] . VAPB shows an higher expression in spinal cord compared to other tissues according to the Genotype-Tissue Expression (GTEx) project [7] and it is detected at mRNA and protein level quite exclusively in human and murine lumbar spinal cord motor neurons [2, 6] . Correlation analyses for VAPB with DNAJA1, HSF1, HSPA8 and HYOU1 are not shown in Fig. 5 due to lack of post mortem material to complete the analyses.

***Quantitative real-time PCR (qPCR)***

qPCR was performed using the Power Up™ SYBr™ Green Master Mix with a primer concentration of 6 μM. An AriaMx Real-time PCR System was used with the following cycling conditions: 95 **°** C for 10 minutes, and then 35 cycles of 95 **°**C for 30 s, Tm **°**C (of gene of interest) for 30 s, 72 **°**C for 1 minute, followed by 95 **°**C for 1 minute, 63 **°**C for 30 s, and 95 **°**C for 30 s. Ct values were normalized to the housekeeping gene beta actin. We compared the expression of GAPDH and Actin in a subset of samples and we found that these two reference genes showed a strong correlation.

***Western blot analysis***

Sections from frozen tissue blocks were cut (15 µm) on a cryostat (Bright Instruments), homogenized and lysed with lysis buffer (20nM Tria-HCl, 137mM NaCl, 10% glycerol, 1% NP40, 2nM EDTA). Proteins were separated as described in Montibeller *et al.* [1] . Briefly, with 10% or 12% SDS-PAGE based on the molecular weight of the protein investigated. Briefly, appropriate volumes of 4x Laemnli SDS sample buffer (v/v, Alfa Aesar) were then added. Homogenised tissues were warmed at 95°C for 5 min and equal protein amounts were separated from the different samples by 10% SDS-PAGE and blotted onto nitrocellulose membranes. Transfer efficiency was checked with Ponceau (Sigma) staining. Blots, blocked in 1% milk/PBS-Tween-20 (PBST), were probed with specific antibodies using dilutions and incubation times suggested by the manufacturer. Immunodetection was performed using the ECL reagents (Thermo Scientific). Densitometry quantification of the bands was performed using ImageJ software (National Institute of Health Bethesda, MD, USA). Antibodies used were anti-HERPUD1 (Cell Signalling Technology), anti-PDIA6 (Abcam), anti-HSPA5/GRP78 (Proteintech), anti-DNAJC10 (Proteintech), anti-PDIA3 (Proteintech), anti-PDIA4 (Proteintech) and anti-β actin (Proteintech).

***Immunohistochemical staining and analysis of VAPB, DNAJC10 and PDIA6 in frontal cortex***

The primary antibodies used for immunohistochemistry were rabbit polyclonal anti-VAPB (Sigma Aldrich HPA-13144; Immunogen: Vesicle-associated membrane protein-associated protein B/C recombinant protein epitope signature tag (PrEST); dilution 1:500), rabbit polyclonal anti-DNAJC10 (Proteintech 13101-1-AP; Immunogen: DNAJC10 fusion protein Ag3737; dilution 1:100), and rabbit polyclonal anti-PDIA6 (Abcam ab11432; Immunogen: Synthetic peptide corresponding to Human PDIA6 aa 476-492; dilution 1:250). Formalin-fixed, paraffin-embedded tissue blocks from the frontal cortex of control and FTLD cases were used for immunohistochemical staining. Tissues were placed were in an oven for 1h, dewaxed with 2 x xylene, and rehydrated with decreasing concentrations of ethanol (3x 100%, 90%, 70%). The tissues were then incubated with hydrogen peroxide for 30 minutes. Antigen retrieval was carried out with citrate buffer pH6, by a series of heating with microwave and cooling stages. The slides were washed with TBS-0.05% Tween buffer for anti-PDIA6, or TBS for anti-VAPB and anti-DNAJC10, and then blocked using the BLOXALL^®^ blocking solution from VECTASTAIN^®^ Elite^®^ ABC Universal PLUS kit for 10 minutes. The slides were then incubated with Normal Horse Serum (2.5%) for 20 minutes. The primary antibodies were diluted in the normal horse serum and incubated overnight at 4°C. The slides were then incubated with pre-diluted horse anti-mouse/rabbit IgG, biotinylated secondary antibody, provided from the kit, followed by VECTASTAIN Elite ABC reagent. Each slide was incubated with the DAB reagent for 25 seconds until the appropriate intensity developed. For counterstaining, haematoxylin was added for 1 minute and 10 seconds. Finally, the slides were dehydrated with series of increasing concentration of ethanol (70%, 90%, 3x 100%), and then 3x xylene, and mounted using DPX. For quantification of protein staining, 5 out of the 10 cases per condition per antibody were selected for quantification. The most optimal slides were selected according to quality of staining, tissue damage and background staining. Representative areas of immunohistochemically stained samples were captured using light microscope and the software ImagePro at 20X magnification. Two regions per slide were captured, one at cortical layer 3 and one at cortical layer 5 in the grey matter. One image at 4X magnification was captured for reference. The captured samples were quantified using the software ImageJ, where the intensity of DAB signal against haematoxylin stain was measured with the “colour deconvolution” plug-in.

***Statistical analysis***

Prism and R were used to draw graphs. All data were checked for the presence of outliers by performing the graphPad ROUT test. For the gene expression analyses, D’Agostino-Pearson test was used to test the normality of the data distribution. Sample size was described in the legend of each figure. Where qPCR and correlation analysis was carried out in two batches, the means and the housekeeping gene correlation of each control group were compared and, if different, a normalization factor was applied.

***References***

[1] **Montibeller L, de Belleroche J**. Amyotrophic lateral sclerosis (ALS) and Alzheimer’s disease (AD) are characterised by differential activation of ER stress pathways: focus on UPR target genes. *Cell Stress Chaperones* 2018; 23; 897–912.

[2] **Anagnostou G, Akbar MT, Paul P, et al.** Vesicle associated membrane protein B (VAPB) is decreased in ALS spinal cord. *Neurobiol. Aging* 2010; 31; 969–985.

[3] **Spaethling JM, Na Y-J, Lee J, et al.** Primary Cell Culture of Live Neurosurgically Resected Aged Adult Human Brain Cells and Single Cell Transcriptomics. *Cell Rep.* 2017; 18; 791–803.

[4] **Habib N, Avraham-Davidi I, Basu A, et al.** Massively parallel single-nucleus RNA-seq with DroNc-seq. *Nat. Methods* 2017; 14; 955–8.

[5] **Lake BB, Chen S, Sos BC, et al.** Integrative single-cell analysis of transcriptional and epigenetic states in the human adult brain. *Nat. Biotechnol.* 2018; 36; 70–80.

[6] **Qiu L, Qiao T, Beers M, et al.** Widespread aggregation of mutant VAPB associated with ALS does not cause motor neuron degeneration or modulate mutant SOD1 aggregation and toxicity in mice. *Mol. Neurodegener.* 2013; 8; 1.

[7] **GTEx Consortium**. Human genomics. The Genotype-Tissue Expression (GTEx) pilot analysis: multitissue gene regulation in humans. *Science* 2015; 348; 648–60.

[8] **Paul P, Murphy T, Oseni Z, et al.** Pathogenic effects of amyotrophic lateral sclerosis-linked mutation in D-amino acid oxidase are mediated by D-serine. *Neurobiol. Aging* 2014; 35; 876–85.

[9] **D’Erchia AM, Gallo A, Manzari C, et al.** Massive transcriptome sequencing of human spinal cord tissues provides new insights into motor neuron degeneration in ALS. *Sci. Rep.* 2017; 7; 10046.

**
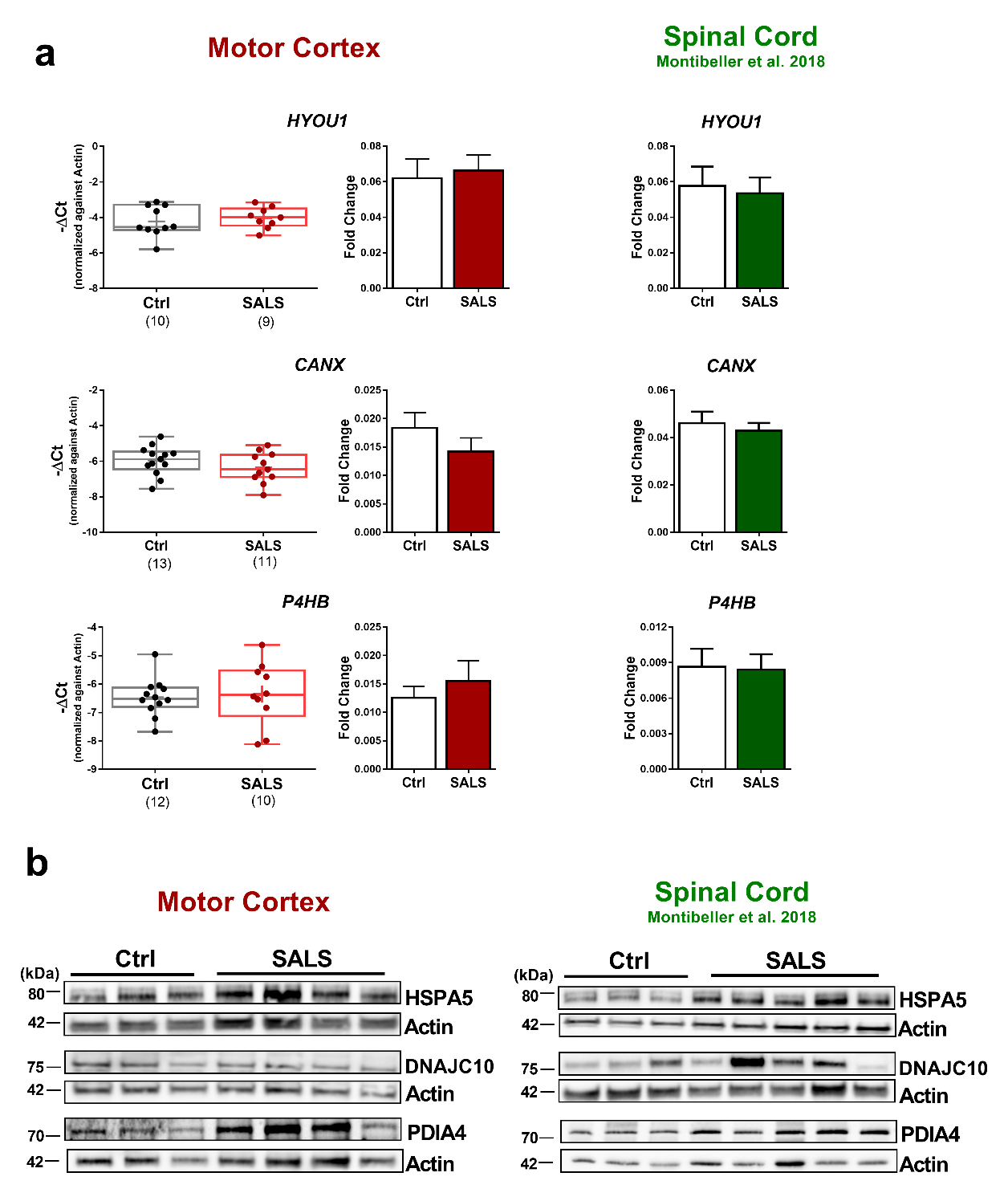
*SI Figures***

**Figure S1. Gene expression and protein level of ER stress genes in motor cortex and spinal cord derived from SALS cases.** (a) mRNA expression analysis of *DNAJC3*, *HYOU1*, *CANX* and *P4HB* in motor cortex of healthy individuals (Ctrl, black) and sporadic cases of amyotrophic lateral sclerosis (SALS, red). Box plot and bar plot are representations for the same samples. Median, maximum and minimum values were used to represent the data as box and whiskers; mean was shown as “+” inside the box. Means and SEMs were used to represent the data in the bar plot. The dots represent individual samples. (b) Representative western blots are shown for HSPA5, DNAJC10 and PDIA4 in motor cortex and spinal cord of healthy individuals (Ctrl) and SALS cases (SALS). Gene and protein expression in spinal cord samples (SALS, green) were obtained from Montibeller and de Belleroche [1] . The numbers under the graphs represent the number of samples analysed. SALS, sporadic amyotrophic lateral sclerosis; Ctrl, control. According to D’Agostino and Pearson normality test, all data are normally distributed. Unpaired *t*-test was used; *p<0.05; **p<0.01.

**
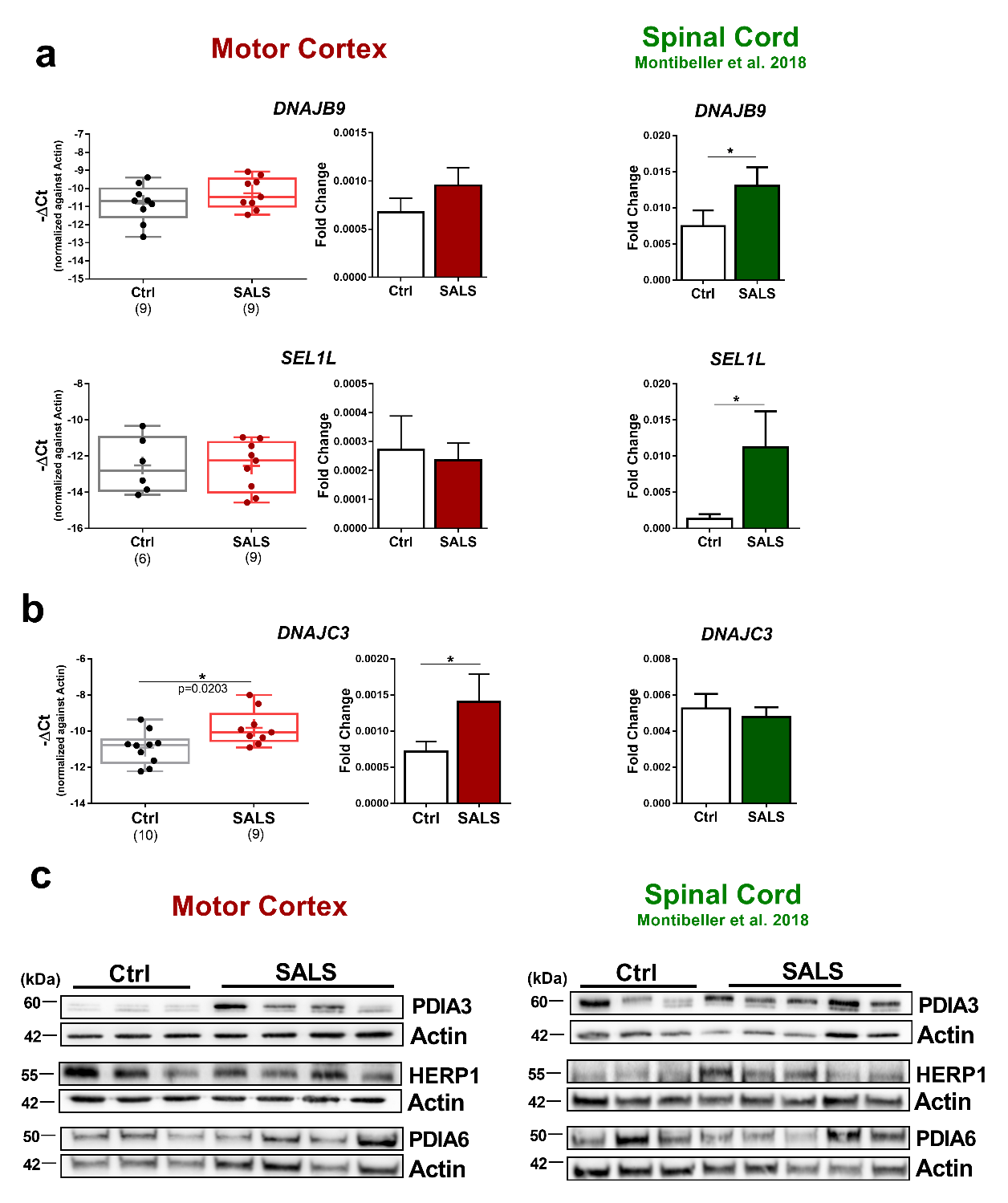
**

**Figure S2. Divergent changes in gene expression and protein level of ER stress genes in motor cortex and spinal cord derived from SALS cases.** (a) mRNA expression analysis of *DNAJB9* and *SEL1L* in motor cortex of healthy individuals (Ctrl, black) and sporadic cases of amyotrophic lateral sclerosis (SALS, red). Box plot and bar plot are representations for the same samples. Median, maximum and minimum values were used to represent the data as box and whiskers; mean was shown as “+” inside the box. Means and SEMs were used to represent the data in the bar plot. The dots represent individual samples. (b) mRNA expression analysis of *DNAJC3* in motor cortex of healthy individuals (Ctrl, black) and sporadic cases of amyotrophic lateral sclerosis (SALS, red). Box plot and bar plot are representations for the same samples. Median, maximum and minimum values were used to represent the data as box and whiskers; mean was shown as “+” inside the box. Means and SEMs were used to represent the data in the bar plot. The dots represent individual samples. (c) Representative western blots are shown for PDIA3, HERPUD1 (HERP1) and PDIA6 in motor cortex and spinal cord of healthy individuals (Ctrl) and SALS cases (SALS). Gene and protein expression in spinal cord samples (SALS, green) were obtained from Montibeller and de Belleroche [1] . The numbers under the graphs represent the number of samples analysed. SALS, sporadic amyotrophic lateral sclerosis; Ctrl, control. According to D’Agostino and Pearson normality test, all data are normally distributed. Unpaired *t*-test was used; *p<0.05; **p<0.01.

**
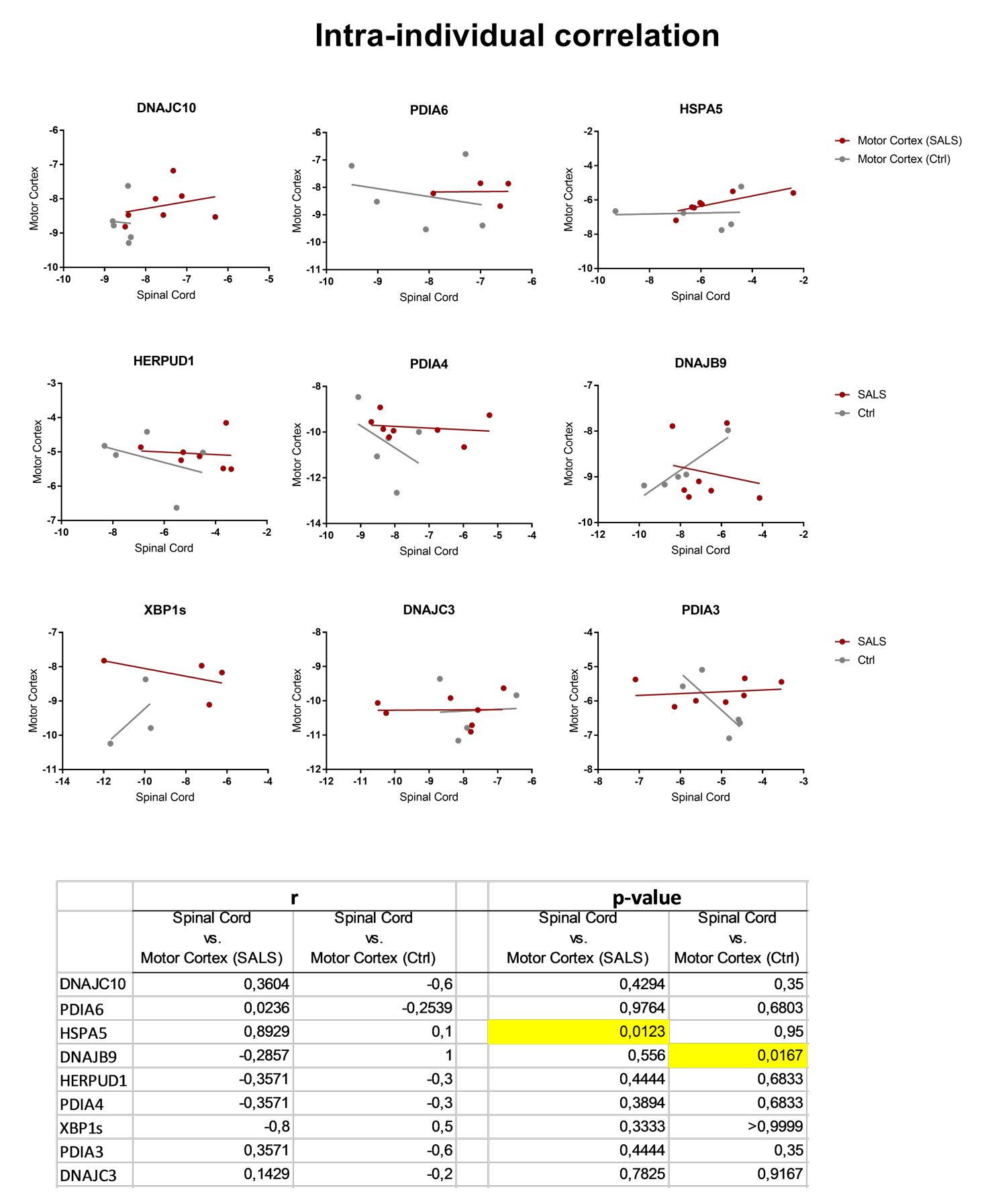

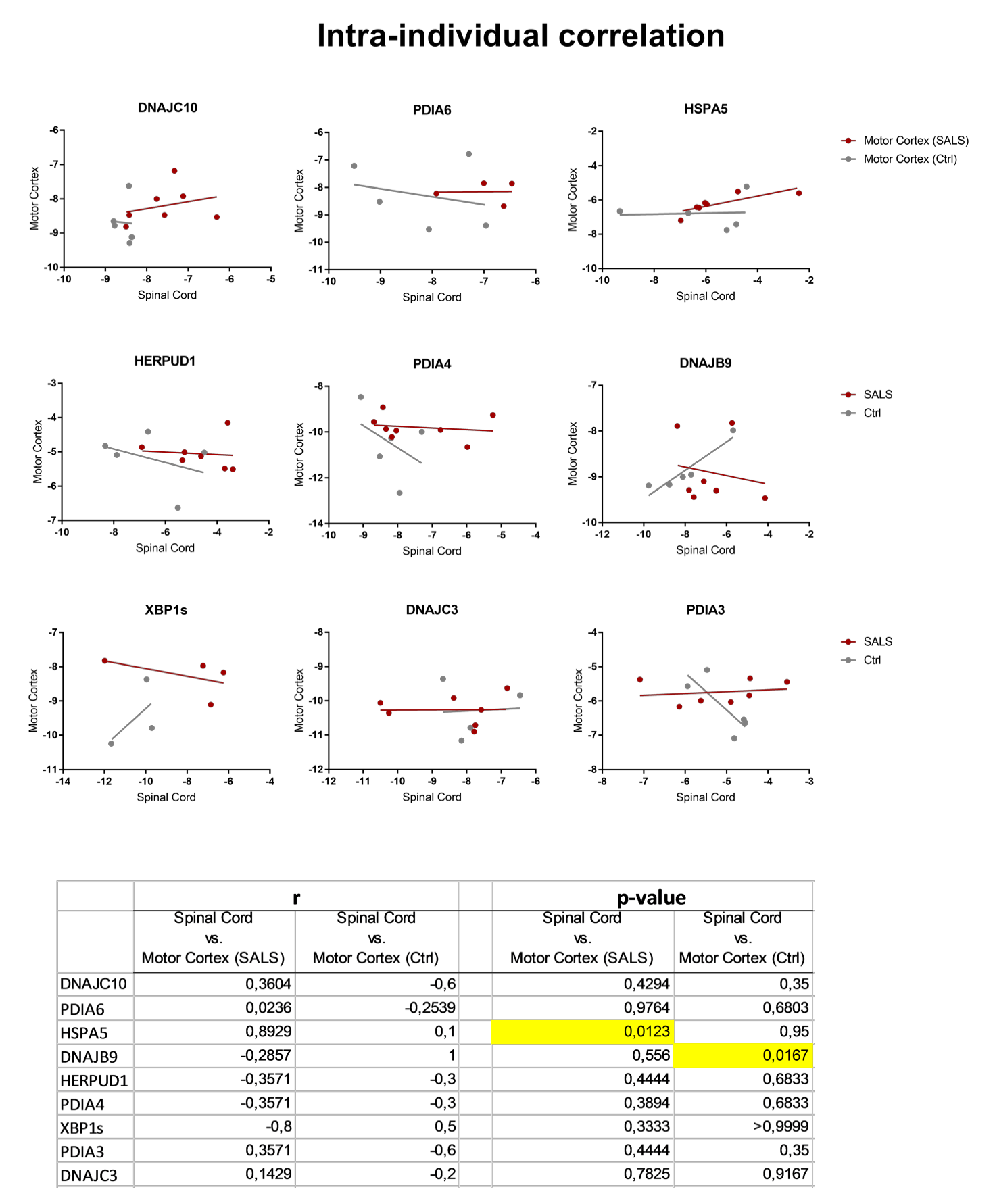
**

**Figure S3. Correlation analyses between motor cortex and spinal cord of the** expression of 9 representative genes**.** Correlation analyses between motor cortex and spinal cord expression of 9 representative genes in 7 SALS and 5 healthy cases. The dots represent individual samples which were divided in healthy controls (grey) and SALS cases (red) and the respective trending lines. Spearman correlation was used since the data were not sampled from a Gaussian distribution in both motor cortex and spinal cord. Ctrl, control; SALS: sporadic amyotrophic lateral sclerosis.

**
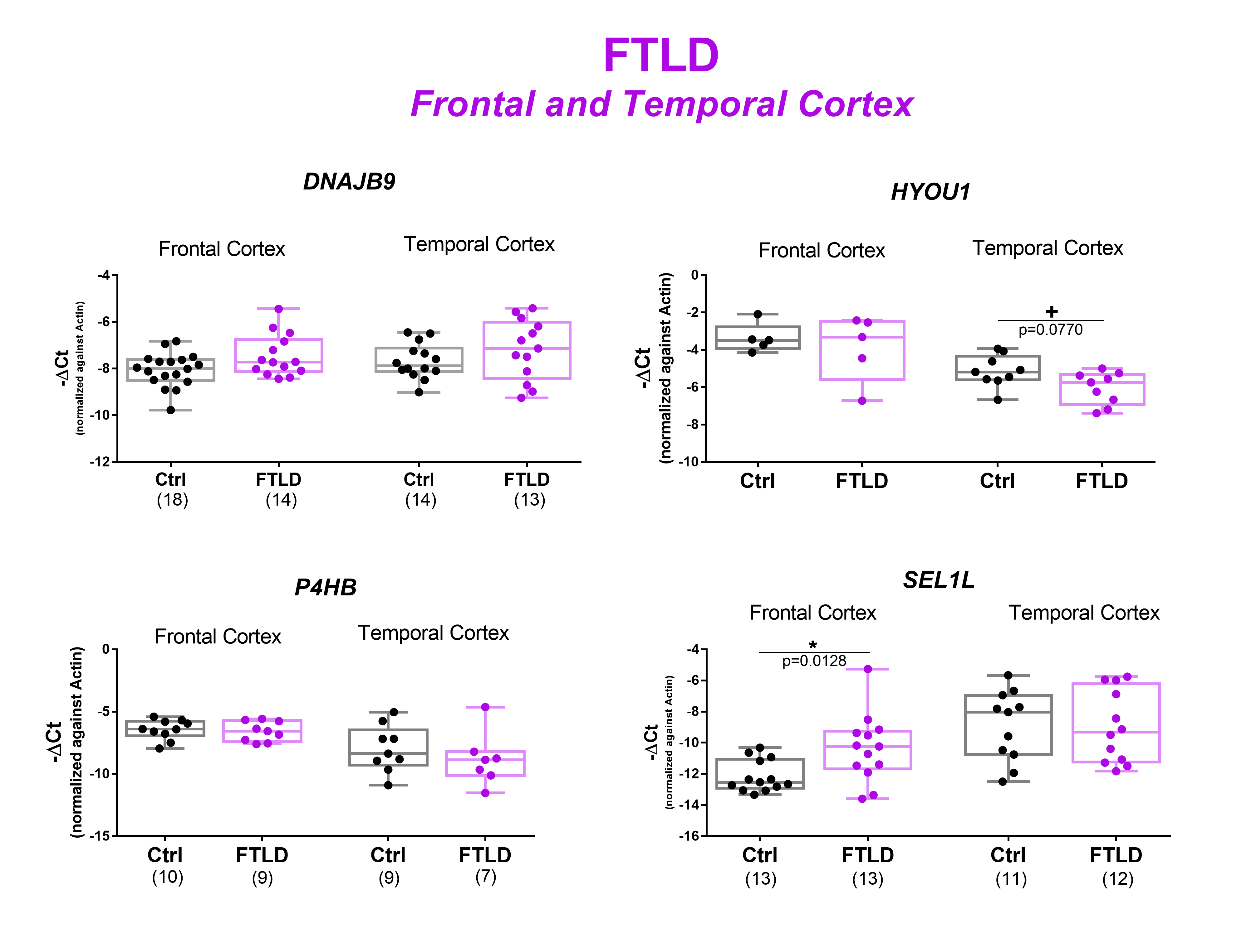
**

**Figure S4. Gene expression of ER stress genes in frontal and temporal cortex derived from FTLD cases.** mRNA expression analysis of *DNAJB9*, *HYOU1*, *P4HB* and *SEL1L* in frontal and temporal cortex of healthy individuals (Ctrl, black) and frontotemporal lobar degeneration cases (FTLD, orange). Box plot and bar plot are representations for the same samples. Median, maximum and minimum values were used to represent the data as box and whiskers; mean was shown as “+” inside the box. Means and SEMs were used to represent the data in the bar plot. The dots represent individual samples. The numbers under the graphs represent the number of samples analysed. FTLD, frontotemporal lobar degeneration; Ctrl, control. According to D’Agostino and Pearson normality test, all data are normally distributed. Unpaired *t*-test was used; +p<0.1; *p<0.05; **p<0.01; ***p<0.001.

**
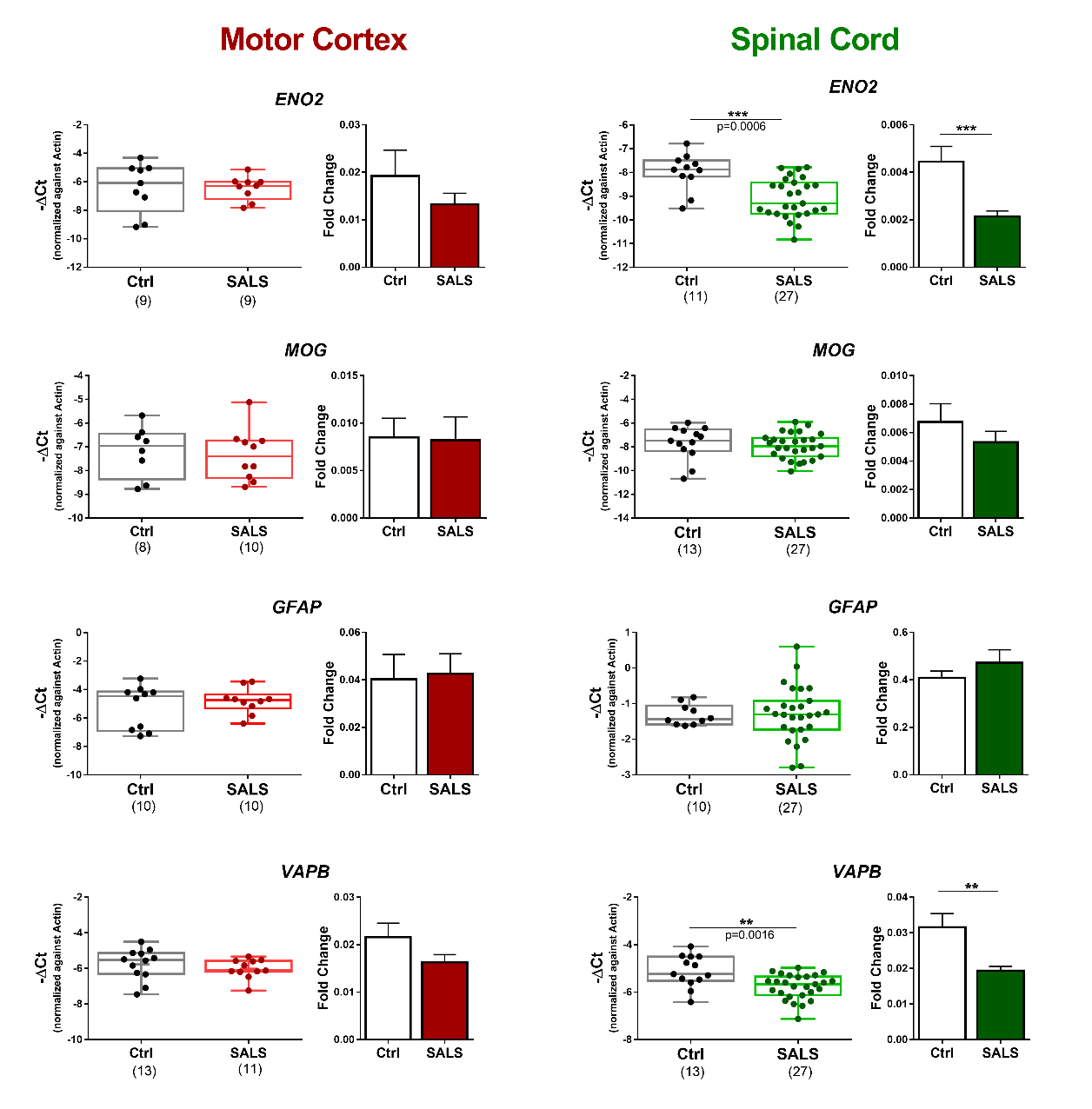
**

**Figure S5. Gene expression of cell type markers in motor cortex and spinal cord derived from SALS cases.** mRNA expression analysis of *ENO2*, *MOG*, *GFAP* and *VAPB* in motor cortex and spinal cord of healthy individuals (Ctrl, black) and sporadic cases of amyotrophic lateral sclerosis (SALS; motor cortex: red, spinal cord: green). Box plot and bar plot are representations for the same samples. Median, maximum and minimum values were used to represent the data as box and whiskers; mean was shown as “+” inside the box. Means and SEMs were used to represent the data in the bar plot. The dots represent individual samples. We considered *GFAP* as a marker for astrocytes, *MOG* for oligodendrocytes, *ENO2* for neurons and VAPB for motor neurons. The numbers under the graphs represent the number of samples analysed. SALS, sporadic amyotrophic lateral sclerosis; Ctrl, control. According to D’Agostino and Pearson normality test, all data are normally distributed. Unpaired *t*-test was used; +p<0.1; *p<0.05; **p<0.01.

**
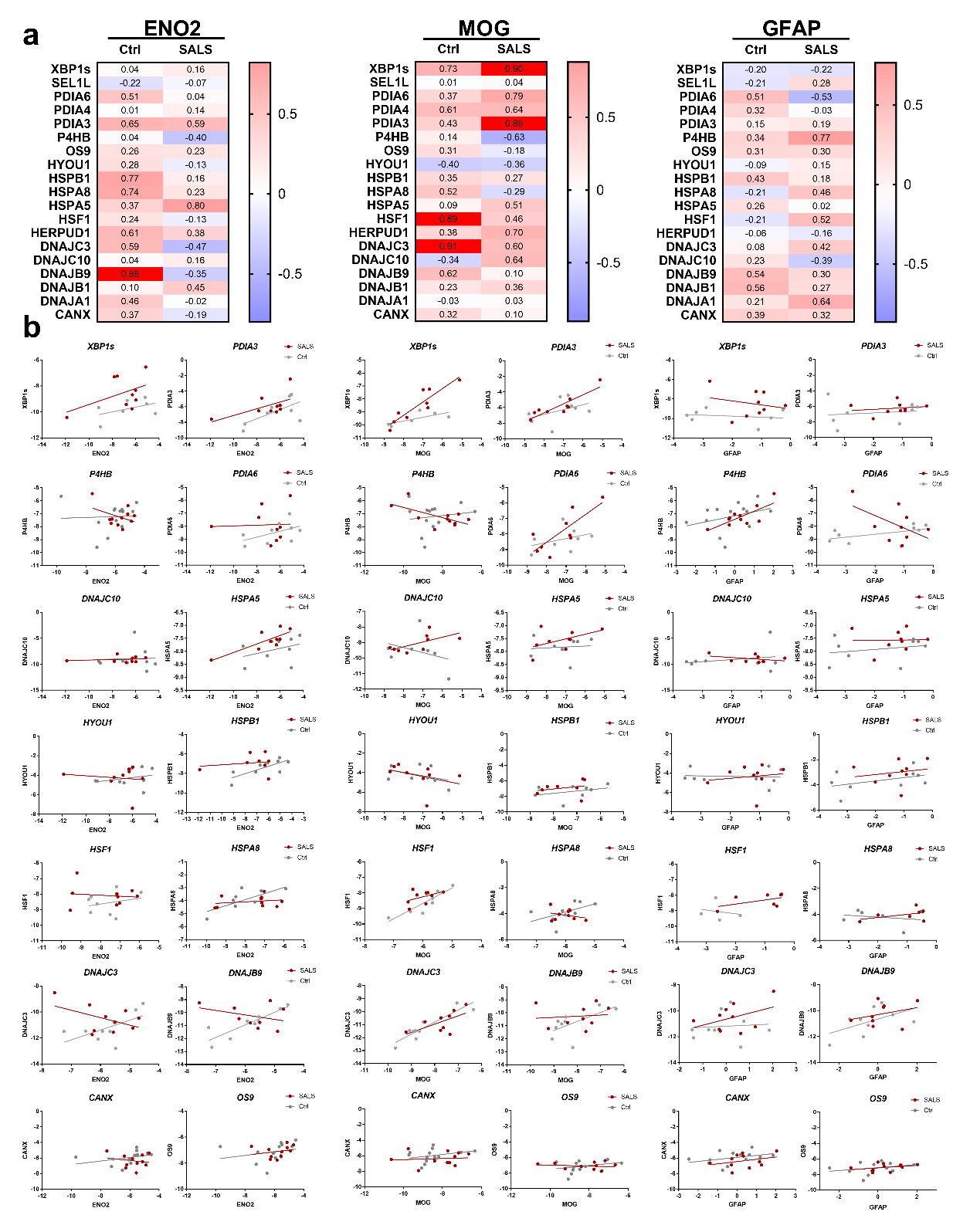
**

**Figure S6. Correlation analyses between cell type markers and UPR-HSR genes in motor cortex derived from healthy and SALS cases.** (a) Heat map of the r values derived from correlation analyses between UPR-HSR and cell type markers genes in motor cortex of healthy individuals (Ctrl) and sporadic cases of amyotrophic lateral sclerosis (SALS). Each box of the heat maps, divided in two columns (Ctrl, SALS), corresponds to a cell type marker specified above and each raw corresponds to a specific gene. Pearson correlation was used when data were normally distributed according to D’Agostino and Pearson normality test; Spearman correlation was used only for SEL1L since the data were not sampled from a Gaussian distribution in both motor cortex and spinal cord. Ctrl, control; SALS: sporadic amyotrophic lateral sclerosis. The coloured bar indicates the range of intensity values for each gene in the heat maps. (b) Correlation analyses of representative genes for each cell type marker are shown under the heat maps. The dots represent individual samples which were divided in healthy controls (grey) and SALS cases (red) and the respective trending lines.

**
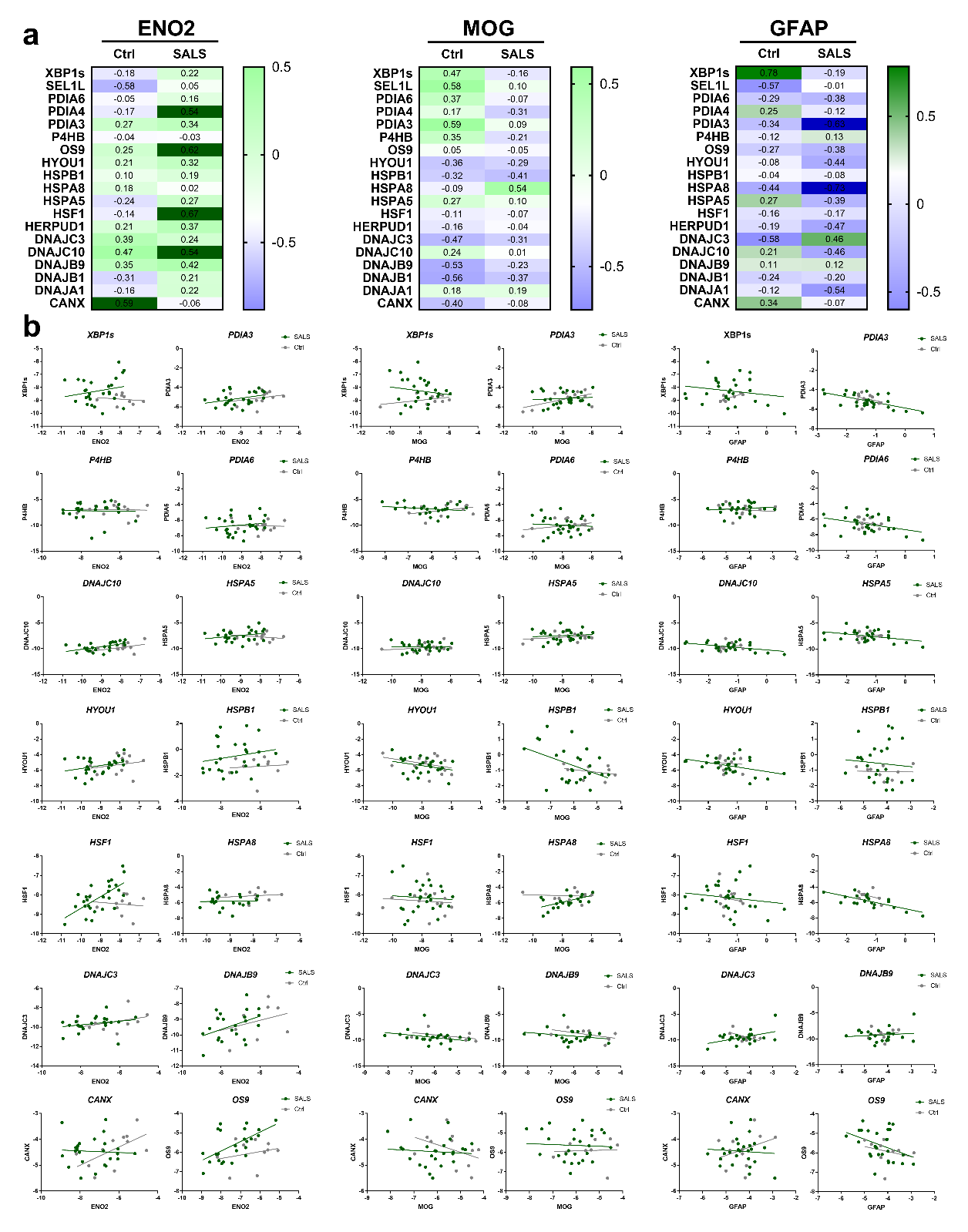
**

**Figure S7. Correlation analyses between cell type markers and UPR-HSR genes in spinal cord derived from healthy and SALS cases.** (a) Heat map of the r values derived from correlation analyses between UPR-HSR and cell type markers genes in spinal cord of healthy individuals (Ctrl) and sporadic cases of amyotrophic lateral sclerosis (SALS). Each box of the heat maps, divided in two columns (Ctrl, SALS), corresponds to a cell type marker specified above and each raw corresponds to a specific gene. Pearson correlation was used when data were normally distributed according to D’Agostino and Pearson normality test; Spearman correlation was used only for SEL1L since the data were not sampled from a Gaussian distribution in both motor cortex and spinal cord. Ctrl, control; SALS: sporadic amyotrophic lateral sclerosis. The coloured bar indicates the range of intensity values for each gene in the heat maps. (b) Correlation analyses of representative genes for each cell type marker are shown under the heat maps. The dots represent individual samples which were divided in healthy controls (grey) and SALS cases (green) and the respective trending lines.

**
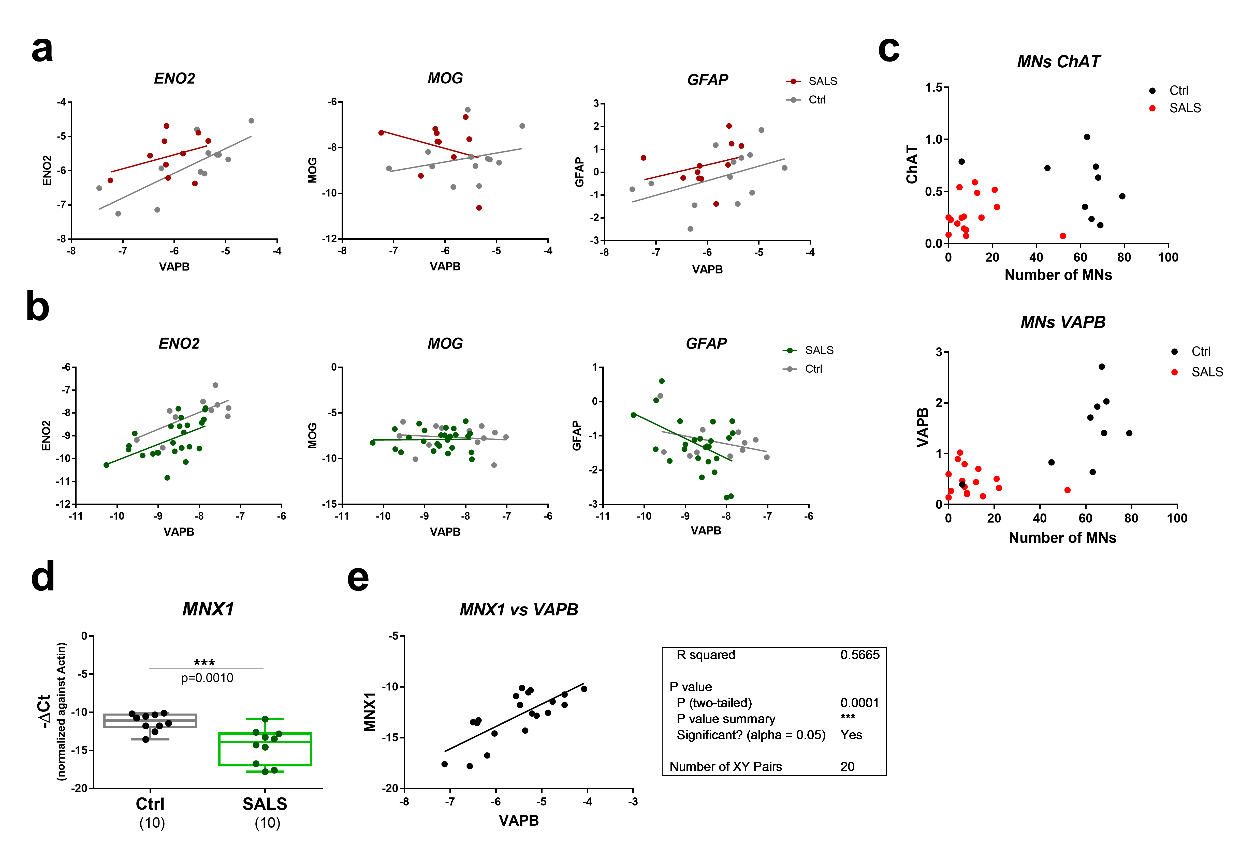
**

**Figure S8. Correlation analyses between motor neuron marker VAPB and cell type markers in motor cortex and spinal cord derived from SALS cases.** (a-b) Correlation analyses of cell-type specific markers with the putative motor neuron marker VAPB. The dots represent individual samples which were divided in healthy controls (grey) and SALS cases in motor cortex (red) and spinal cord (green) with the respective trending lines. Cell type specific markers used were as follow, ENO2 for neurons, MOG for oligodendrocytes, GFAP for astrocytes. (c) Quantification of immunohistochemical analyses of VAPB and ChAT in lumbar spinal cord derived from SALS and healthy cases. Data derived from Paul *et al.*[8] . (d) Gene expression of a well-known motoneuron marker, MNX1, in spinal cord of SALS and healthy individuals. (e) Correlation analysis between VAPB and MNX1 in spinal cord of SALS and healthy individuals. The enclosed table reports the values for the coefficient of determination (r^2^=0.5666), *P* value (p=0.0001) and data nmber analysed (number of XY Pairs).

**
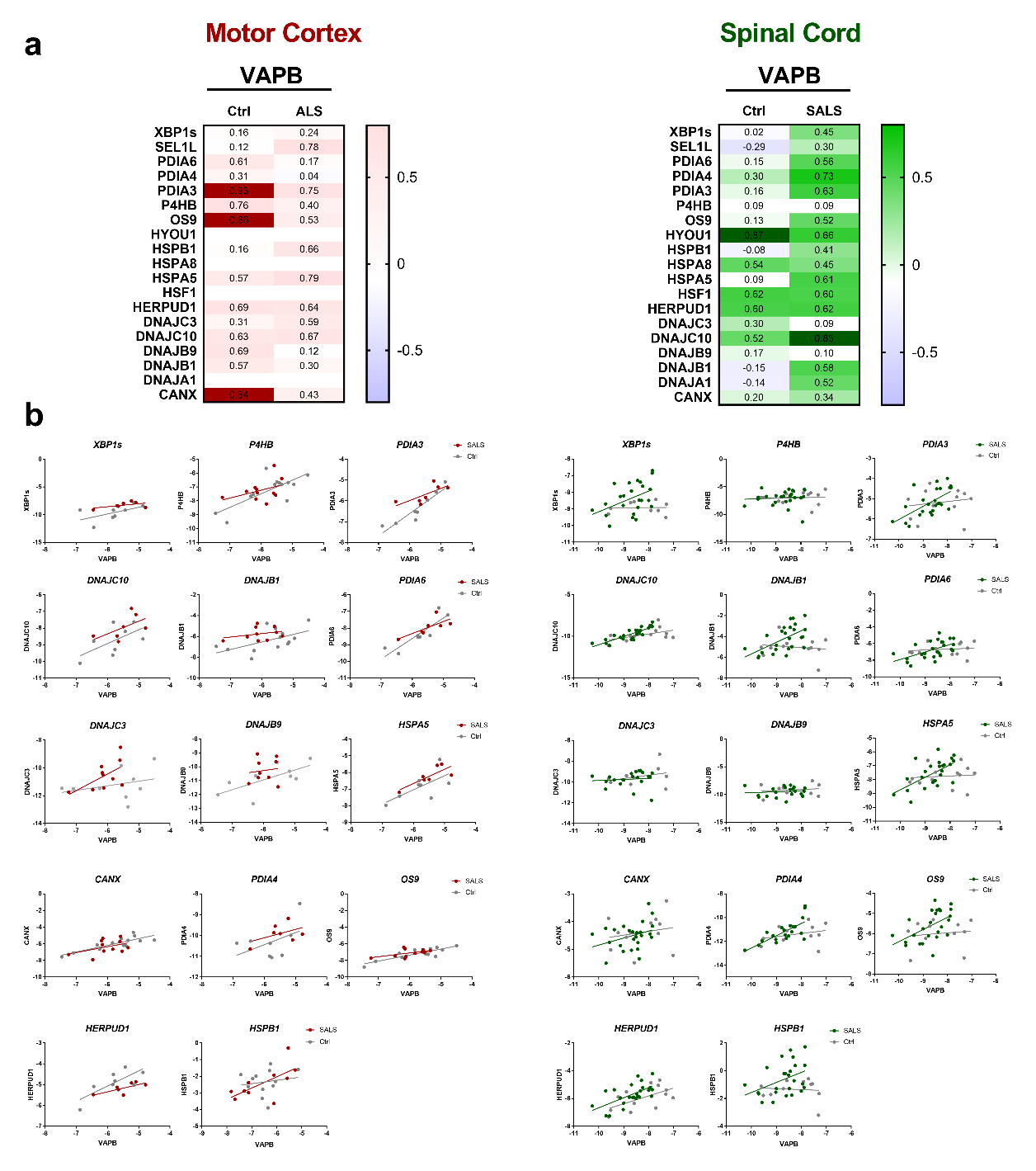
**

**Figure S9. Correlation analyses between putative motor neuron marker VAPB and UPR-HSR genes in spinal cord and motor cortex derived from healthy and SALS cases.** (a) Heat map of the r values derived from correlation analyses between UPR-HSR and putative motor neuron marker VAPB in motor cortex (red) and spinal cord (green) of healthy individuals (Ctrl) and sporadic cases of amyotrophic lateral sclerosis (SALS). Each box of the heat maps, divided in two columns (Ctrl, SALS), corresponds to the motor neuron marker VAPB and each raw corresponds to a specific gene. Pearson correlation was used when data were normally distributed according to D’Agostino and Pearson normality test; Spearman correlation was used only for SEL1L since the data were not sampled from a Gaussian distribution in both motor cortex and spinal cord. Ctrl, control; SALS: sporadic amyotrophic lateral sclerosis. The coloured bar indicates the range of intensity values for each gene in the heat maps. (b) Correlation analyses of representative genes for the motor neuron marker are shown under the heat maps. The dots represent individual samples which were divided in healthy controls (grey) and SALS cases (motor cortex: red; spinal cord: green) and the respective trending lines.

***SI Tables***

| **Table S1**. Clinical details of patient samples, including gender, age at death, and post-mortem delay. | | | |
| --- | --- | --- | --- |
| **Group** | **n** | **Age at death (years)**  median (range) | **Post-mortem delay (hours)**  median (range) |
| Controls | 13 *male = 7* | 67 (20 – 94) | 10.75 (3 – 35) |
|  | *female = 5* |  |  |
|  |  |  |  |
| SALS | 10 *male = 8* | 66.5 (48 – 82) | 14 (7.5 – 60) |
|  | *female = 2* |  |  |

| **Table S2**. List of Primers | | | | | | | |  |
| --- | --- | --- | --- | --- | --- | --- | --- | --- |
| **Gene** | **Primers** | | |  | | | **Tm°** |  |
|  | **FORWARD** | | | **REVERSE** | | |  |  |
| ACTIN B | CTGGAACGGTGAAGGTGACA | | | AAGGGACTTCCTGTAACAATGCA | | | 57 |  |
| MOG | CTTGAAGAGCTACGAAATCC | | | CAAATTGGAATGTCCCCATAG | | | 57 |  |
| ENO2 | GTCCATAGAGAAGATCTGGG | | | CTAAGTAACGCTGTTTGTCTC | | | 57 |  |
| GFAP | CTGCAGATTCGAGAAACCAG | | | GACTCCTTAATGACCTCTCCA | | | 59 |  |
| HSPB1 | TGGTGATCTCGTTGGACTGC | | | CTTCACGCGGAAATACACGC | | | 59 |  |
| HSF1 | AACAGAAAGTCGTCAACAAG | | | CTATACTTGGGCATGGAATG | | | 55 |  |
| DNAJB1 | TTAAGAGAGATGGCTCTGATG | | | CTTTGAATACGACGGGTATC | | | 59 |  |
| DNAJA1 | GGTAAACTTTCCTGAGAATGG | | | CATCATCCTCATATGCTTCTC | | | 55 |  |
| HSPA8 | CTATCACTAATGACAAGGGC | | | GAATTCTTGGATGACACCTTG | | | 55 |  |
| VAPB | CCAATAGTGTCTAAGTCTCTGAG | | | GTCCATCTTCTTCCTTGAACTG | | | 56 |  |
|  |  | | |  | | |  |  |
| **Table S3**. Expression of “C3HC4-type RING finger domain binding” GO term members in RNA-Seq from D’Erchia *et al*. [9] | | | | | | | | |
| **Gene** | | **log2FoldChange** | **padj** | | **regulat** | **signif** | | |
| KCNH2 | | -0,770941 | 0,00153315 | | Down | √ | | |
| PINK1 | | -0,431614 | 0,01445307 | | Down | √ | | |
| DNAJA1 | | -0,362663 | 0,05369269 | | Down | √ | | |
| HSPA1A | | -0,172142 | 0,82882195 | | Down |  | | |
| HSPA1B | | -0,925155 | 0,14628058 | | Down |  | | |
| HSPA8 | | -0,513930 | 0,01866356 | | Down | √ | | |
|  | |  |  | |  |  | | |
| D’Erchia, A. M. *et al.* Massive transcriptome sequencing of human spinal cord tissues provides new insights into motor neuron degeneration in ALS. *Sci. Rep.* **7**, 10046 (2017). | | | | | | | | |
